# Multifunctional Scaffolds for Assembling Cancer-Targeting Immune Stimulators Using Chemoselective Ligations

**DOI:** 10.3389/fchem.2019.00113

**Published:** 2019-03-06

**Authors:** Anne C. Conibear, Karine Thewes, Nadja Groysbeck, Christian F. W. Becker

**Affiliations:** Faculty of Chemistry, Institute of Biological Chemistry, University of Vienna, Vienna, Austria

**Keywords:** ligation, click chemistry, orthogonal ligations, protein conjugation, peptide functionalization, peptide-polymer conjugates

## Abstract

Chemoselective ligations allow chemical biologists to functionalise proteins and peptides for biomedical applications and to probe biological processes. Coupled with solid phase peptide synthesis, chemoselective ligations enable not only the design of homogeneous proteins and peptides with desired natural and unnatural modifications in site-specific locations but also the design of new peptide and protein topologies. Although several well-established ligations are available, each method has its own advantages and disadvantages and they are seldom used in combination. Here we have applied copper-catalyzed azide-alkyne “click,” oxime, maleimide, and native chemical ligations to develop a modular synthesis of branched peptide and polymer constructs that act as cancer-targeting immune system engagers (ISErs) and functionalised them for detection in biological systems. We also note some potential advantages and pitfalls of these chemoselective ligations to consider when designing orthogonal ligation strategies. The modular synthesis and functionalization of ISErs facilitates optimisation of their activity and mechanism of action as potential cancer immunotherapies.

## Introduction

The use of antibodies for cancer therapy takes advantage of the specific binding of antibodies to their targets and has led to the introduction of several antibody-based cancer therapeutics into the clinic (Scott et al., 2012; Sliwkowski and Mellman, 2013). Although antibodies have high specificity, limited tissue penetration and difficulties in generating large amounts of homogeneous antibody products cost-effectively initially restricted their use, and a wide range of antibody fragments, antibody-drug conjugates and antibody-like molecules have been developed to address these challenges (Casi and Neri, 2015; Weiner, 2015; Haußner et al., 2017). In particular, peptides and peptide-polymer conjugates can mimic the functions of large folded proteins such as antibodies and have the advantage that they are synthetically accessible and readily modified and optimized for specific biological functions (Ahrens et al., 2012; Fosgerau and Hoffmann, 2015; Gross et al., 2015; Conibear et al., 2017a). Furthermore, peptides, polymers, proteins and small molecules can be ligated together to generate highly specific multifunctional compounds for biomedical applications (Jewett and Bertozzi, 2010; Schumacher and Hackenberger, 2014; Agarwal and Bertozzi, 2015; Merten et al., 2015; Bondalapati et al., 2016).

**Figure 1:**
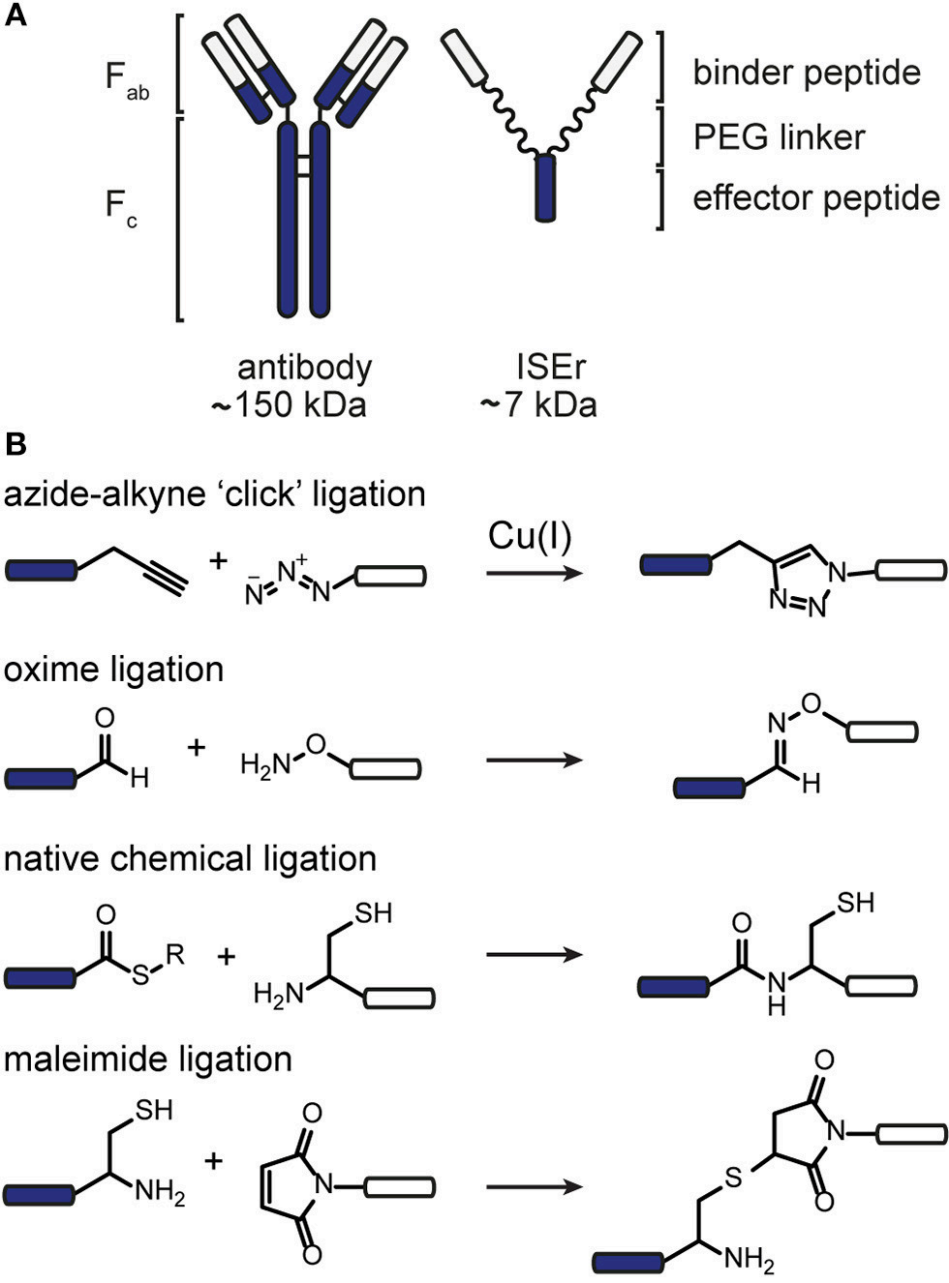
Chemoselective ligations for functionalization of peptide-polymer conjugates named ISErs. **(A)** ISErs mimic the functions of monoclonal antibodies in that they bind specifically to a target receptor and ellicit an immune response. However, ISErs are entirely synthetic and comprise an effector peptide conjugated to two or more binder peptides *via* a polyethylene glycol (PEG) linker. **(B)** Chemoselective ligation reactions used to functionalise ISErs in this work. Blue and white bars represent peptide segments, polymer chains, or label/tag molecules.

Chemoselective ligation reactions have enabled the generation of numerous molecular tools and probes. The diverse applications of chemoselective ligations are possible due to the ready availability of molecules bearing the respective functional groups, their compatibility with biological systems, and aqueous buffers in particular, and their generally stable and non-toxic products (Patterson et al., 2014; Patterson and Prescher, 2015) Amongst the most commonly used ligation reactions for biomedical applications are copper-catalyzed azide-alkyne “click” (CuAAC), oxime, maleimide and native chemical (NCL) ligations, illustrated in Figure 1. The functional groups involved in these reactions can be installed in peptides using commercially available building blocks that are stable to solid phase peptide synthesis conditions. Choosing a ligation strategy when designing a particular conjugate, however, is not always straightforward, and extensive optimisation often needs to be carried out for each application (Patterson and Prescher, 2015) Nevertheless, combining chemoselective ligations can provide access to sophisticated highly-functionalized biomolecules with precisely designed properties. For example, Galibert et al. have demonstrated the use of oxime, CuAAC and maleimide ligations in various combinations to functionalize a cyclic peptide scaffold with Arg-Gly-Asp (RGD)-moieties, as well as carbohydrate, nucleic acid and a fluorophore (Galibert et al., 2009, 2011). In this work, we have applied the chemoselective reactions above for the functionalization of peptide-polymer scaffolds that mimic the functions of natural antibodies, enabling modular syntheses and rapid optimisation and modification.

We have previously reported the development of a prototype innate immune system engager (ISEr), a peptide-polymer conjugate that mimics the functions of antibodies in targeting cancer cells and eliciting an immune response (Brehs et al., 2017) ISErs are synthesized entirely by solid phase peptide synthesis (SPPS) and comprise two or more “binder” peptides that specifically target receptors overexpressed on cancer cells and are conjugated *via* polyethylene glycol (PEG) linkers to an “effector” peptide that stimulates an immune response (Figure 1) (Brehs et al., 2017). To explore the cancer cell specificity and immune stimulatory activity of ISErs, we have generated variants bearing two or more “binders” targeting the same or different receptors and have also used them for targeting cytotoxic drug molecules to cancer cells (Conibear et al., 2017b, 2018a). Further development and optimisation of the ISEr concept and its application to other types of cancer cells requires investigation of additional binder and effector peptides, combinations of binder and effector peptides and labeling of the ISErs to detect them in cells and tissues.

Here we describe how we have used chemoselective ligation strategies and combinations thereof to functionalise ISErs for biomedical applications. The ability to attach various binder peptides and labels to ISErs chemoselectively increases the modularity of the synthesis, the affinity and selectivity of ISErs for cancer cells and their ease of detection in biological contexts. We also highlight some of the advantages and disadvantages of the chemoselective ligation strategies and discuss points to consider in selecting a ligation reaction, as well as how to optimize these reactions and pairs of orthogonal reactions for peptide functionalization.

## Results and Discussion

### Increasing the Modularity of the Synthesis Using Chemoselective Ligations

**Figure 2:**
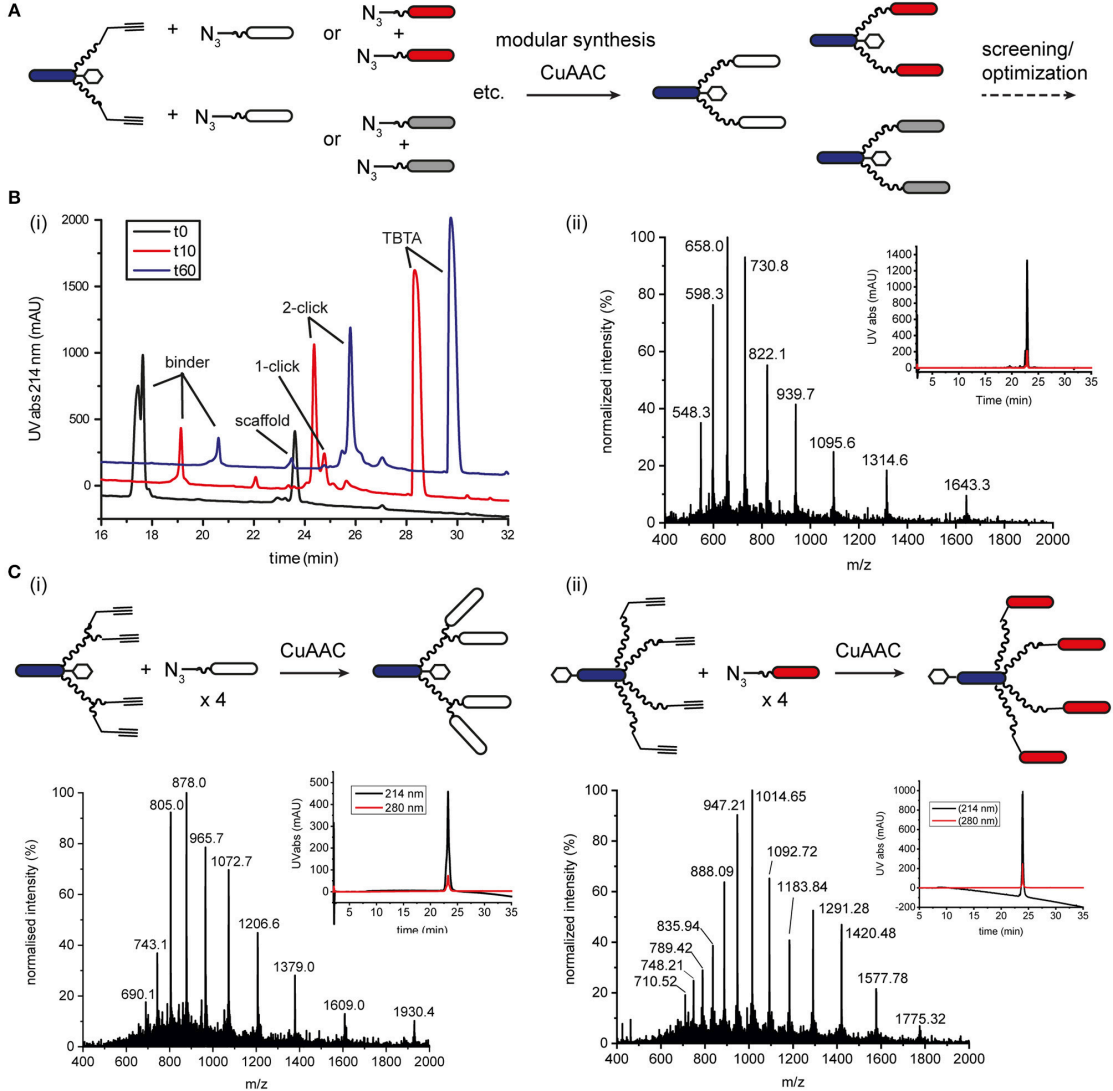
CuAAC ligation to increase the modularity of ISEr synthesis. **(A)** A single alkyne-bearing effector-PEG_27_ scaffold can be ligated to various azide-bearing binder peptides to generate a series of ISErs with different receptor-binding properties for screening and optimization. Binder and effector peptides are represented by differently colored bars, biotin by a white hexagon and PEG chains by wavy lines. **(B)** (i) Time course of the CuAAC ligation between the effector-PEG_27_ scaffold and an integrin α_3_β_1_ targeting binder peptide, monitored by RP-HPLC with UV detection at 214 nm. The HPLC traces are offset by 1.5 min on the x-axis and 100 mAU on the y-axis. The reaction mixture was sampled at 0, 10 and 60 min. (ii) MS and analytical HPLC of the purified CuAAC ligation product, MW_calc_: 6567.9 Da, MW_obs_: 6567.0 Da **(C)** Synthesis, MS and analytical HPLC of tetravalent ISErs bearing (i) two PEG_27_ chains and four integrin α_3_β_1_ targeting binder peptides, MW_calc_: 9646.8 Da, MW_obs_: 9645.5 Da or (ii) four PEG_27_ chains and four EphA2-binding peptides, MW_calc_: 14195.6 Da, MW_obs_: 14196.0 Da.

We sought to develop a modular synthesis of ISErs that would enable the attachment of several different binder peptides to the same peptide-polymer scaffold using chemoselective ligations, so that multiple binders could be tested rapidly. We therefore used the CuAAC ligation to attach the binder peptides to the ends of the PEG_27_ linkers (Figure 2A; Rostovtsev et al., 2002; Tornøe et al., 2002). The widely-used CuAAC ligation is generally fast, selective, compatible with aqueous environments and many azide- and alkyne-functionalised building blocks are commercially available (Kolb and Sharpless, 2003; Meldal and Tornøe, 2008). The Cu(I)-catalyzed cycloaddition between an azide and a terminal alkyne (Figure 1) yields a 1,4-disubstituted 1,2,3-triazole that is stable in biological systems and is similar in length to a peptide bond. Although Cu(I) can be used directly in the form of CuBr or CuI, it is less stable than Cu(II) and therefore the Cu(I) catalyst is often generated *in situ* from CuSO_4_ and a reducing agent such as sodium ascorbate. Several ligands are available to stabilize the Cu(I) species and to reduce oxidative damage of proteins (Meldal and Tornøe, 2008).

The ISEr peptide-polymer scaffold was synthesized by SPPS as described previously with a L-propargyl glycine (alkyne functionality) on the end of each PEG_27_ chain (Conibear et al., 2018a). The short N-formylated “effector” peptide fMIFL (Rot et al., 1987) bore a glycine spacer and two lysine residues to which the PEG_27_ chains were coupled *via* the side chains (Figure S1). As α-carbonyl alkynes have been reported to be more reactive than alkyl-substituted alkynes (Meldal and Tornøe, 2008). we initially coupled propiolic acid to the N-termini of the PEG_27_ chains as the alkyne functionality. Although coupling of the propiolic acid appeared to be successful and several coupling conditions were tested, many side products were observed, possibly involving addition of benzotriazole or azabenzotriazole anions to the activated alkyne (Massif et al., 2012). We therefore proceeded with Fmoc-L-2-propargylglycine, which coupled efficiently to the PEG_27_ chains and, as it is an amino acid, also allows for incorporation of the alkyne moiety at any position in the binder peptide. Several binder peptides were synthesized by SPPS bearing an azidolysine residue (azide functionality), separated from the main peptide chain by a short PEG_3_ linker (Figure 2A and Figures S2–S5). Analytical data, yields and approximate times for synthesis and purification are given in the Supplementary Information.

We tested several conditions to optimize the CuAAC ligation, with different copper sources, ligands and solvents. Initially, the CuAAC ligation was attempted with the alkyne-bearing effector-PEG scaffold on resin, employing CuI as the catalyst, a nitrogen base (2,6-lutidine or diisopropylamine) and the purified azide-bearing binder peptide in solution. In principle, CuI can be used without a reducing agent, but might require a nitrogen base to form the copper-acetylide complex (Rostovtsev et al., 2002). Although the desired triazole-linked ISEr product was observed in some cases, the reaction depended on the length of the PEG chain, with the shorter PEG_4_ reacting more successfully than the longer PEG_27_. Furthermore, the solubility of CuI and stability of the Cu(I) species proved problematic and therefore the solution-phase CuAAC ligation was chosen and optimized for the modular synthesis of ISErs. Nevertheless, on-resin CuAAC ligations have been widely used, especially for intramolecular cyclisation and stapling reactions (Ingale and Dawson, 2011; Lau et al., 2015).

Optimisation of the CuAAC ligation in solution involved screening several copper sources, ligands, reducing agents, temperatures, solvents and reagent concentrations, as summarized in Table 1, which shows a selection of the conditions tested. All reactions were carried out between a purified effector-PEG_27_ alkyne-bearing scaffold and various purified binder peptides targeting different cell surface receptors overrepresented on cancer cells. Use of CuI was partially successful with diisopropylethylamine (DIPEA) as a nitrogen base but, as CuI is not soluble in water or THF, it was difficult to find a suitable solvent mixture for this reaction. Moreover, several side products were observed, probably involving iodination over the prolonged reaction times (12–20 h), as has previously been reported (Goyard et al., 2012, 2014) so we proceeded with *in situ* generation of the Cu(I) species. In this strategy, Cu(II) is reduced to Cu(I) with a reducing agent such as ascorbate in the presence of a stabilizing ligand (Meldal and Tornøe, 2008). We found that the presence of a ligand was necessary for the reaction to proceed and that it was essential to mix the copper and ligand tris[(1-benzyl-1*H*-1,2,3-triazol-4-yl)methyl]amine (TBTA) before addition of the reducing agent. Using sodium ascorbate as the reducing agent in large excess greatly increased the rate of the reaction. Although Cu(I) is theoretically a catalyst, we found that several equivalents were required for efficient and complete reaction, especially in the case of the cysteine-containing α_3_β_1_ integrin-targeting binder peptide (“B9”), where we added additional batches of the copper-ligand mixture during the reaction. As ligand, we further explored tris[(1-hydroxypropyl-1*H*-1,2,3-triazol-4-yl)methyl]amine (THPTA), a water-soluble analog of TBTA, and TentaGel-supported TBTA, which should facilitate separation of the ligand after the reaction. No product was observed with THPTA and, although some product was observed with the polymer-supported TBTA, the reaction was slow and incomplete, so we proceeded with TBTA in a water/DMF mixture. We obtained the best results with TBTA (tris[(1-benzyl-1*H*-1,2,3-triazol-4-yl)methyl]amine) (Chan et al., 2004) as a ligand to stabilize Cu(I), which was formed *in situ* from Cu(II) using sodium ascorbate. As shown in Figure 2B, the reaction was monitored by LC-MS and showed formation of first the “one-click” and then the “two-click” ligation product (Figure 2B).

**Table 1:** Optimisation of solution-phase CuAAC ligation conditions.

**Binder**	**Cu source**	**Additives**	**Solvent(s)**	**Comments**
B2	CuI (0.5 equivalents)	DIPEA (20 equivalents)	ACN/H_2_O (80:20)	Product observed, reaction incomplete, M+125, M+250 (iodination of Tyr?)
B2	CuI (2 equivalents)	DIPEA (20 equivalents)	THF/ACN (27:73)	Product observed after overnight reaction, reaction incomplete
B2	CuSO_4_ (4 equivalents)	TBTA (4 equivalents), ascorbic acid (20 equivalents)	H_2_O/DMSO (50:50)	Product observed, oxidation of Met
B3	CuSO_4_ (4 equivalents)	TBTA (4 equivalents), ascorbic acid (20 equivalents)	DMF/H_2_O (84:16)	Product observed, reaction incomplete after 21 h. Some oxidation of Met
B3	CuSO_4_ (4 equivalents)	TBTA (4 equivalents), sodium ascorbate (40 equivalents)	DMF/H_2_O (83:17)	Reaction complete in < 5 min. Good solvent mixture and large excess of sodium ascorbate keeps Cu reduced.
B9	CuSO_4_ (2 × 3 equivalents)	TBTA (2 × 3.5 equivalents), sodium ascorbate (30 equivalents)	DMF/H_2_O (84:16)	Product observed but reaction incomplete after 80 min. Binder peptide with cysteine residues reacts slowly.
B9	CuSO_4_ (6 equivalents)	THPTA (30 equivalents), sodium ascorbate (55 equivalents)	H_2_O	No product observed, decomposition of starting materials after extended reaction time (17 h).
B9	CuSO_4_ (36 equivalents)	TentaGel-TBTA (10 equivalents), sodium ascorbate (260 equivalents)	DMF/H_2_O (85:15)	Product observed but reaction incomplete after 72 h. Separation of product easier but CuAAC would require further optimization with on-resin TBTA.
B9	CuSO_4_ (6 equivalents)	TBTA (6.5 equivalents), sodium ascorbate (50 equivalents)	DMF/H_2_O (81:19)	Reaction almost complete after 1 h. Large excess of Cu(I) required for peptides containing cysteine.

*B2: H-YLFSVHWPPLKA-OH*.

*B3: H-PQNSKIPGPTFLDPH-OH*.

B9: H-cdGY(3-NO_2_)GHypNc-NH_2._

The rate of CuAAC ligation varied widely with different binder peptides, ranging from 5 min to over an hour to reach completion. We found that the reaction rate depended on the amino acid composition of the binder peptides; peptides containing cysteine residues reacted much slower than those without and peptides containing arginine residues were modified by excess ascorbate in the reaction mixture. In the former case, free cysteine thiols likely complexed with the copper catalyst and we found that the reaction rate and purity was improved if disulfide bonds in the peptide were formed prior to the CuAAC ligation. The modification of arginine by dehydroascorbate was explored in more detail and could be minimized by using a smaller excess of ascorbate and monitoring the reaction carefully to avoid prolonged exposure of the peptides to the dehydroascorbate (Conibear et al., 2016). Overall however, we found the CuAAC ligation to be a fast and reliable means of increasing the modularity of peptide-polymer scaffold synthesis and the products were purified from excess starting materials and TBTA by RP-HPLC to give the desired bivalent triazole-linked ISErs (Figure 2B). Although the rates of the CuAAC ligations varied for the different binders, overall preparation times were approximately the same as most time is required for HPLC purification and analysis. We were able to generate ISErs with binder peptides targeting integrin α_3_β_1_ (CD49c/VLA-3), (Yao et al., 2009a,b) integrin α_v_β_6_, (Zhou et al., 2004) c-Met/HGFR, and EphA2 (Koolpe et al., 2002) receptors, as well as their scrambled counterparts as controls, all using the same effector-PEG_27_ scaffold.

In addition to increasing the modularity of ISEr synthesis, we further used CuAAC ligations to generate multivalent ISErs to explore avidity and affinity effects of cancer cell binding (Conibear et al., 2018a). Using a similar strategy to that described for the bivalent ISErs above, we synthesized an effector-PEG_27_ scaffold with either two L-propargyl glycine residues on the ends of each of two PEG_27_ chains, or four PEG_27_ chains each with one L-propargyl glycine residue (Figures S6, S7). Tetravalent ISErs were then generated by carrying out four CuAAC ligations simultaneously to attach four binder peptides. As shown in Figure 2C, this yielded the desired tetravalent products after HPLC purification. Similarly to the bivalent examples, we found that prolonged exposure to the CuAAC reaction reagents was detrimental and that the reaction was most efficient for peptides that did not contain cysteine residues. Furthermore, the tetravalent product sometimes co-eluted with the excess TBTA, complicating the purification process and resulting in decreased yields. TBTA is poorly water-soluble and so could be partially removed from the reaction mixture by precipitation after dilution with water. Although there are several water-soluble ligands that we could have explored (Besanceney-Webler et al., 2011; Ekholm et al., 2016) carrying out the CuAAC ligation in DMF (in which TBTA is highly soluble) also allows the peptide reagents to be dissolved at high concentrations, increasing the reaction rate and yield. In general, although the reactions appeared to reach completion by LC-MS analysis, we achieved isolated yields of 30–40% for the CuAAC ligations. An alternative strategy, which would avoid the need for copper and TBTA, is the strain-promoted azide-alkyne click (SPAAC) ligation developed in the Bertozzi group (Agard et al., 2004). This method has found wide application in biological systems where the use of copper could cause toxicity. However, the large and hydrophobic alkyne moiety is not easily compatible with the conditions employed in SPPS so we did not pursue this strategy for synthetic ISErs.

**Figure 3:**
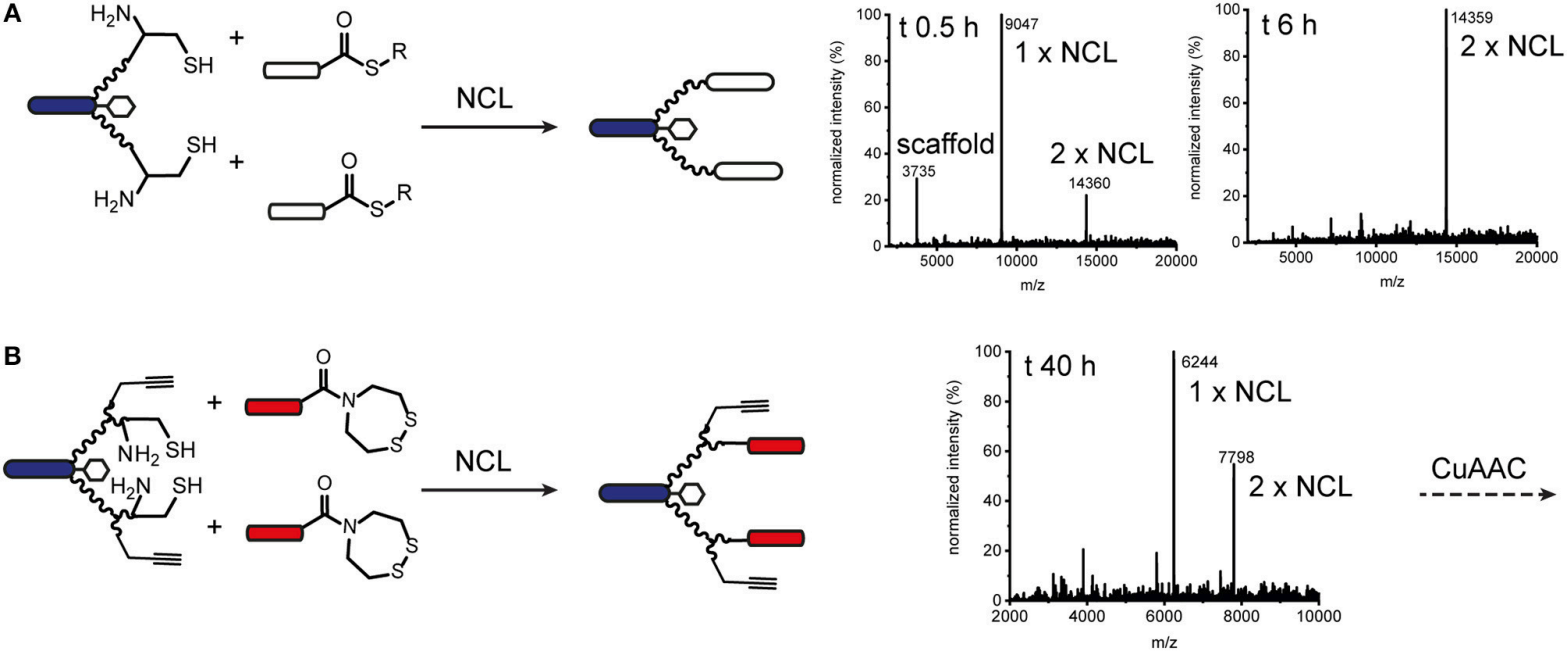
Native chemical ligation for modular synthesis of bivalent and tetravalent bispecific ISErs. **(A)** NCL of test peptide thioesters to effector-PEG_27_ scaffold bearing two N-terminal cysteine residues. Deconvoluted mass spectra after 0.5 h and 6 h ligation showing formation of the desired double ligation product. **(B)** NCL of EphA2 binding peptides bearing SEA thioester precursors to effector-PEG_27_ scaffold bearing two N-terminal cysteine residues and two propargyl glycine residues. Deconvoluted mass spectra after 40 h ligation showing only partial formation of the desired double ligation product.

We also explored native chemical ligation (NCL) for attaching thioester-functionalised binder peptides to an effector-PEG_27_ scaffold bearing cysteine residues on the ends of the PEG_27_ chains (Figure 3 and Figure S8). NCL is widely used for the synthesis and semi-synthesis of proteins as it can be carried out under mild aqueous conditions and yields a native peptide bond at the ligation site (Dawson et al., 1994; Conibear et al., 2018b). Similarly to the CuAAC ligations, the efficiencies of the native chemical ligations varied with the binder peptide thioesters. Whereas a test peptide ligated cleanly and efficiently to the ISEr scaffold (Figure 3A), synthetic epidermal growth factor (EGF) segments ligated slowly and incompletely and were prone to aggregation in the ligation buffer, especially as the peptide chain grew longer with successive ligations (Gell et al., 2017). The efficiency of NCL is dependent on the nature of the C-terminal residue of the thioester fragment and the solubility and length of the peptides. Recent developments in and extensions to NCL, including new methods for generating peptide thioesters, new thiol additives and selenocysteine-mediated ligations, have greatly expanded the scope of NCL (Conibear et al., 2018b), but were not explored extensively for ISErs. For bispecific ISErs generated using two orthogonal ligation strategies, we explored the *bis*(2-sulfanylethyl)amino (SEA) thioester precursor strategy that enables synthesis of the binder peptides by Fmoc-SPPS followed by *in-situ* formation of the C-terminal thioester by N-S-acyl rearrangement under reducing conditions (Ollivier et al., 2010). Synthesis of the effector scaffold bearing handles for both NCL and CuAAC (Figure S9) and binder peptide precursors (Figure S10) proceeded smoothly but ligation at both PEG_27_ termini did not reach completion (Figure 3B). A possible explanation for this could be the lack of an electron-withdrawing group alpha to the carbonyl of the thioester surrogate, which increases reactivity. Although alternative strategies for optimizing the ligation or generating the C-terminal thioesters on the binder peptides are available (Conibear et al., 2018b; Kulkarni et al., 2018) the strategy would have required additional selective protection of the α_3_β_1_ integrin binder, which contains an N-terminal D-cysteine that is important for biological activity (Yao et al., 2009a) so we turned to oxime ligation as an orthogonal alternative to use in combination with CuAAC.

### Generating Multispecific Peptide-Polymer Conjugates Using Orthogonal Chemoselective Ligations

**Figure 4:**
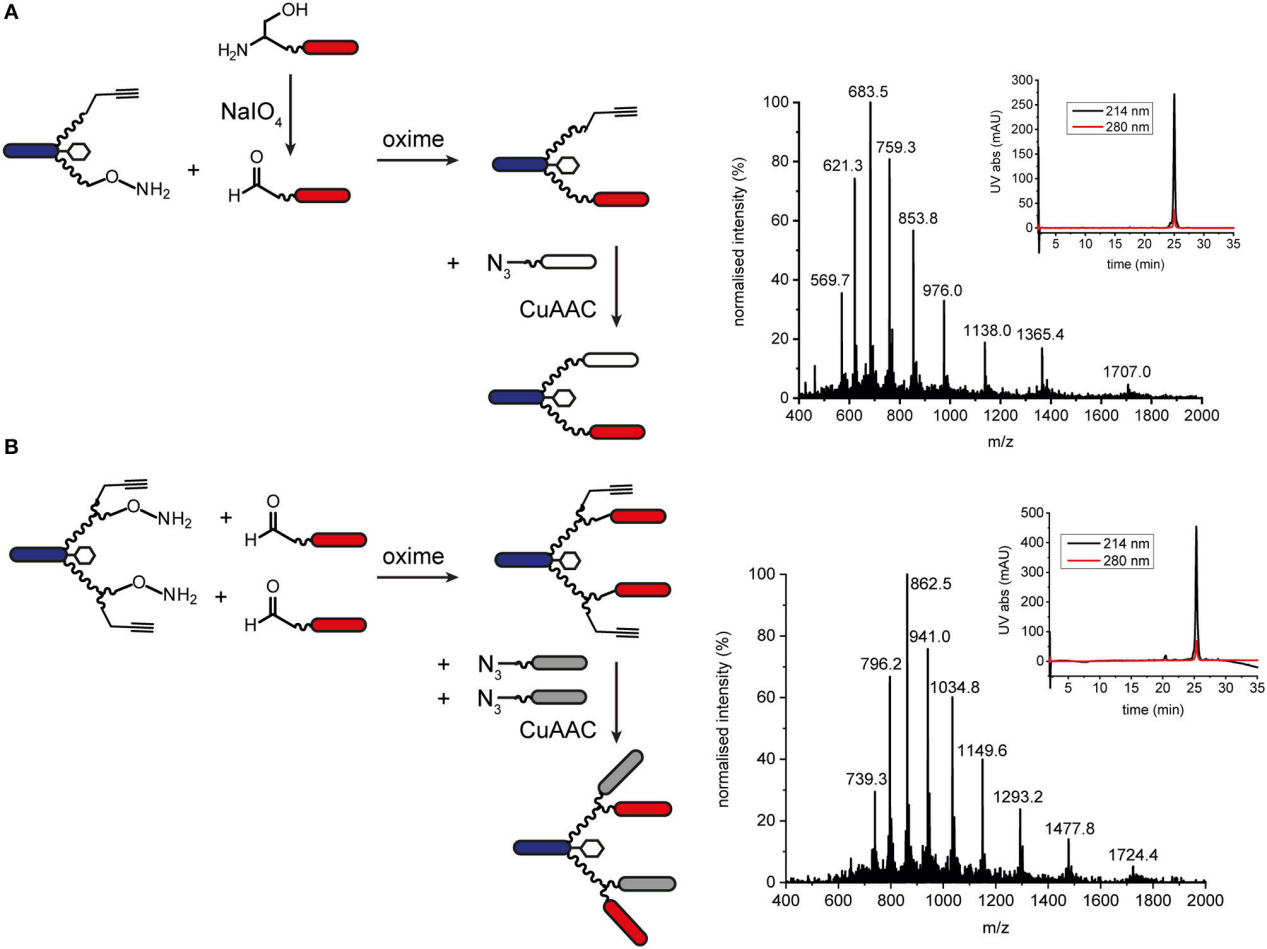
Oxime and CuAAC ligations for modular synthesis of bispecific ISErs. **(A)** An aldehyde-bearing EphA2-binding peptide was generated from the corresponding N-terminal serine-bearing precursor by oxidation using NaIO_4_. This binder peptide was ligated to the effector-PEG_27_ scaffold using oxime ligation and then an azide-bearing integrin α_3_β_1_ binding peptide was ligated using CuAAC ligation. MS and analytical HPLC of the bivalent bispecific ISEr product. MW_calc_: 6819.9 Da, MW_obs_: 6818.5 Da. **(B)** Aldehyde-bearing EphA2 binders were ligated to the effector-PEG_27_ scaffold using oxime ligation followed by azide-bearing integrin α_3_β_1_ binding peptides using CuAAC ligation. MS and analytical HPLC of the tetravalent bispecific ISEr product. MW_calc_: 10335.8 Da, MW_obs_: 10338.5 Da.

Orthogonal chemoselective ligations were used to synthesize peptide-polymer conjugates bearing two different binder peptides targeting different cell surface receptors to increase specificity and affinity. For this strategy, we employed the CuAAC ligation described above for one binder and oxime ligation for the second. The reaction between a hydroxylamine and an aldehyde or ketone under aqueous conditions at slightly acidic pH gives rise to an oxime linkage between the two components (Figure 1; Rose, 1994; El-Mahdi and Melnyk, 2013). As previous experiments had shown that the N-terminal cysteine residue of the α_3_β_1_ integrin binder (“B9”) was important for activity (Yao et al., 2009a) we chose to ligate this binder to the scaffold using CuAAC ligation and the second binder, an EphA2-binding peptide (“B59”), (Koolpe et al., 2002) using oxime ligation. For this strategy, we synthesized scaffolds bearing either an aminooxy acetic acid (Aoa) functionality on one PEG_27_ chain and a propargyl glycine on the other (Figure S11, to give a bivalent bispecific ISEr, Figure 4A) or an aminooxy acetic acid and a propargyl glycine on each PEG_27_ chain (Figure S12, to give a tetravalent bispecific ISEr, Figure 4B). In both cases, we observed side-products with two or no Aoa functionalities that were challenging to purify. Nevertheless, the desired bispecific ISErs were obtained in acceptable purity for bioactivity assays, which showed that the bispecific bivalent ISEr had higher affinity for PC-3 cancer cells (K_d_ = 13.0 ± 2.3 nM) than either of the two monospecific bivalent ISErs (K_d_ = 30.4 ± 10.0 nM and 68.9 ± 19.0 nM) (Conibear et al., 2018a).

We explored several strategies for installing the aldehyde or ketone moiety on the binder peptides. Whereas, a keto-functionality can be installed at either the N- or C-terminus, the aldehyde was generated by periodate oxidation of an N-terminal serine (Geoghegan and Stroh, 1992), separated from the binder peptide by a short PEG_3_ linker. Several methods for increasing the rate and yield of oxime ligations have been published, including catalysis by aniline and by freezing (Dirksen et al., 2006; El-Mahdi and Melnyk, 2013; Agten et al., 2016), however, in our hands, we were able to obtain the best results by reacting an aldehyde with the aminooxy peptide in acetonitrile/water at pH 4.5 in acetate buffer. Several bispecific ISErs were obtained using oxime ligation followed by CuAAC ligation in both bivalent and tetravalent forms (Figure 4). Although the two reactions are theoretically orthogonal, it was necessary to do the oxime ligations first, as the aminooxy functionalities on the scaffold underwent several side-reactions on incubation in DMF with the CuAAC reagents, even in the absence of an azide component. This problem could potentially have been overcome by using a protected aminooxy functionality, such as 1-ethoxyethylidine (Eei) (Foillard et al., 2008; Galibert et al., 2009). The design of the synthesis strategy and choice of ligations was also influenced by the sequences of the binder peptides; as the EphA2 binder already contains an N-terminal serine in the binding sequence, which might affect binding affinity if oxidized to an aldehyde, it was necessary to insert a PEG_3_ linker between this residue and the N-terminal serine that was oxidized to form the aldehyde moiety for oxime ligation (Geoghegan and Stroh, 1992).

### Functionalizing Peptide-Polymer Scaffolds With Tags and Labels for Detection

**Figure 5:**
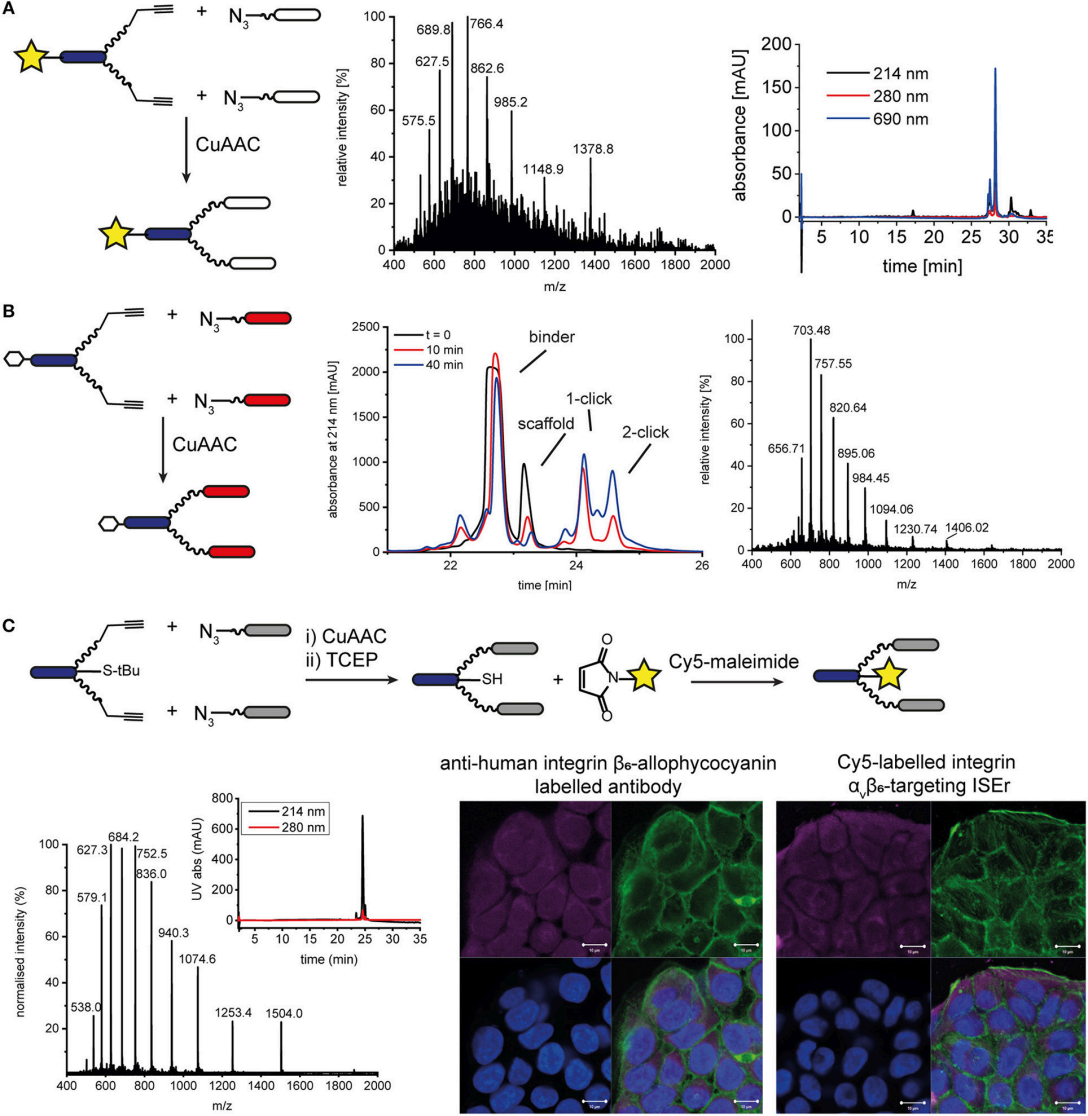
N- and C-terminal labeling of ISEr scaffold for detection. **(A)** A fluorescent dye DY680 (yellow star) was coupled via NHS-ester to the N-terminus of the effector-PEG_27_ scaffold and two integrin α_3_β_1_ binding peptides were ligated using CuAAC ligation. MS and analytical HPLC (with fluorescence detection at 690 nm) traces are shown for the purified product: MW_calc_: 6889.0 Da, MW_obs_: 6890.5 Da. **(B)** CuAAC ligation of cMET binders to N-terminal biotinylated effector-PEG_27_ scaffold. Biotin is represented by a white hexagon. The reaction was monitored by RP-HPLC and the purified product was analyzed by MS: MW_calc_: 9836.4 Da, MW_obs_: 9837.0 Da. **(C)** C-terminal labeling of ISErs using orthogonal chemoselective ligations. Integrin α_v_β_6_-targeting binder peptides were ligated to the effector-PEG_27_ scaffold by CuAAC ligation. After removal of the S-tBu cysteine protecting group with TCEP, the fluorescent dye maleimide was coupled to the free thiol. MS and analytical HPLC trace of purified Cy5-labeled ISEr (MW_calc_: 7513.5 Da, MW_obs_: 7514.0 Da). Fluorescence microscopy image showing binding of a commercial anti-human integrin β_6_-allophycocyanain-labeled antibody (left) and Cy5-labeled ISEr (right) to HT-29 cells, detected at 647 nm (top left, magenta), cell cytoskeleton stained with phalloidin (top right, green), nuclei stained with 4′,6-diamidino-2-phenylindole (DAPI, bottom left, blue) and overlay (bottom right). Scale bar = 10 μm.

In addition to functionalising our peptide-polymer conjugates with varying numbers and arrangements of binder peptides, we have also used chemoselective ligations to functionalise ISErs with biotin tags and fluorescent dyes to enable measurement of their binding affinities and biodistribution. Initially, we synthesized the effector-PEG_27_ scaffold using SPPS and coupled either Fmoc-biotinyl-PEG_2_-OH or a fluorescent dye N-hydroxy succinimide (NHS) ester in place of the N-formyl moiety on the N-terminus of the effector peptide. These biotinylated or fluorescently labeled effector-PEG_27_ scaffolds could then be used in CuAAC ligations as described above. For example, DY680 activated NHS ester (Dyomics GmbH) was coupled to the effector-PEG_27_ scaffold on resin (Figure S13). The resulting DY680-labeled effector-PEG_27_ scaffold was then ligated to two integrin α_3_β_1_ binding peptides *via* CuAAC ligation (Figure 5). Similarly, an N-terminal biotin-labeled effector-PEG_27_ scaffold was ligated to two cMET binding peptides (Figure 5 and Figure S14). Although these N-terminally labeled scaffolds were used to detect binding of the respective ISErs to cancer cells after ligation of the binder peptides, using either indirect detection with streptavidin-FITC (biotin-tagged) or direct fluorescence detection (dye-labeled), the disadvantage was that they lacked effector activity due to the replacement of the N-terminal formyl group with the biotin or dye.

Two strategies were developed to enable labeling of the peptide-polymer scaffolds at the C-terminal end of the effector peptide, which is not involved in either the immune-stimulating or cancer-targeting activity (Figure 1 and Figure S1): In the first strategy, the effector peptide was synthesized on a biotin-linked resin (Novabiochem) to yield the C-terminal biotinylated effector-PEG_27_ scaffold after cleavage; In the second strategy, a maleimide ligation was used as an orthogonal reaction to attach a maleimide-functionalised fluorescent dye to a thiol group (Cy5, sulfo-Cy5, or Alexa-750). Both strategies maintain the cancer cell-binding and immune activating activities of the binder and effector peptides, respectively. Whereas, the first strategy is straightforward and does not require any additional steps, it relies on the compatibility of the chosen dye or tag to SPPS conditions. The C-terminal biotin was used to determine the immune-activating and cancer cell-binding activities of a series of multivalent and multispecific ISErs generated using the CuAAC and oxime ligations described above (Conibear et al., 2018a).

Attachment of a fluorescent dye to the C-terminus by maleimide ligation required synthesis of the effector-PEG_27_ scaffold with a cysteine residue at the C-terminus of the effector peptide (Figure 5 and Figure S15). This was easily achieved by loading the SPPS resin using a sulfonyl *tert*-butyl (S-tBu)-protected cysteine. The CuAAC ligation to attach the binder peptides was carried out prior to the maleimide ligation to attach the dye in order to minimize use of the expensive dyes. However, for binder peptides that contain cysteine residues, the maleimide ligation would need to be carried out first to prevent labeling of cysteine residues in the binder peptide, or would require orthogonal protection of the cysteine residues. After removing the S-tBu protecting group from the cysteine with *tris*(2-carboxyethyl)phosphine (TCEP), the peptide was purified by HPLC and kept at acidic pH under argon to maintain the reduced thiol. The additional HPLC step decreased the overall yield but was necessary to remove TCEP from the solution, as the phosphine can compete with the thiol for reaction with the maleimide, resulting in undesired phosphine-dye adducts (Henkel et al., 2016). If the TCEP was not removed, no maleimide product was formed and we observed formation of a side-product corresponding to an adduct of TCEP and Cy5 maleimide (MW 855 Da). After removing the TCEP, the maleimide-functionalised dye (Cy5, sulfo-Cy5, or Alexa750) was added in degassed water or DMF and the ligation was complete within minutes. For the coupling of Cy5, which is poorly soluble in water, the maleimide coupling was carried out in a mixture of DMF/water 1:3 and satisfactory ligation was observed. After HPLC purification to remove excess dye, the peptide-polymer conjugates were characterized by MS and HPLC with fluorescence detection (Figure 5 and Figure S16) and used for fluorescence microscopy (Figure 5). Although the CuAAC ligation, StBu removal and Cy5 maleimide coupling reactions all reached completion, based on LC-MS analysis, isolated yields ranged from 41 to 88% and resulted in an overall final yield of 2% based on the synthesis scale of the scaffold. This illustrates the benefits of one-pot approaches that avoid the need for some of the purification steps, which are costly in terms of both material and time.

As a more versatile labeling strategy, we synthesized an integrin α_3_β_1_ binding peptide-polymer conjugate and its scrambled counterpart by SPPS, bearing an azidolysine residue at the C-terminus of the effector peptide (Figures S17, S18). This allows for ligation of an alkyne-functionalised fluorescent label to this cysteine-containing ISEr, without orthogonal protection of the cysteine residues. Using CuAAC, we ligated alkyne-functionalised Cy7 to both the integrin α_3_β_1_ binding ISEr and its scrambled counterpart (Figures S19, S20) for *in vivo* biodistribution studies. While this strategy involved attachment of the binder peptides to the scaffold on resin using standard coupling methods, we could also have combined it with the modular synthesis described above by making use of orthogonal CuAAC ligations. An example of such a strategy was recently reported for antibody protein mimics of infliximab (Longin et al., 2018). In this study, a water-soluble derivative of CycloTriVeratrilene bearing three alkyne moieties was semi-orthogonally protected with triethylsilyl or triisopropylsilyl groups. Three cyclic peptide epitopes were then sequentially ligated to the scaffold using CuAAC ligations in a one-pot strategy (Longin et al., 2018).

## Conclusion

We have used combinations of solid phase peptide synthesis and chemoselective ligations to generate a wide variety of functionalised peptide-polymer conjugates with different arrangements of immune-stimulating peptides, PEG linkers and cancer cell-binding peptides, as well as fluorescent labels and tags. The established CuAAC, NCL, oxime, and maleimide ligations were used alone and as orthogonal pairs to develop modular synthetic strategies to access a library of ISErs. Careful consideration, however, was necessary in selecting and combining ligation reactions based on the nature of the binder peptides and the functional groups present. Although the reactions used here have the advantage that they can be used on unprotected peptides, we found that functionalization of the protected constructs on the solid phase was often higher yielding due to the better control over selectivity. Nevertheless, chemoselective reactions allow coverage of a larger chemical space in a shorter time as we were able to develop a wide range of functionalised ISErs for biological activity testing. Overall, this illustrates the versatility and potential of functionalising synthetic peptide-polymer scaffolds using chemoselective ligations.

## Experimental

### General Methods for Peptide Synthesis and Purification

#### SPPS Methods

Effector and binder peptides were synthesized by manual or automated fluorenylmethoxycarbonyl (Fmoc) chemistry. Peptides with a C-terminal amide were synthesized on Rink amide resin, peptides with a C-terminal acid were synthesized on Wang resin and peptides with C-terminal biotin were synthesized on Biotin NovaTag resin (Merck). For initial residue loading of Wang resin, the amino acid (10 equivalents) was stirred with N,N'-diisopropylcarbodiimide (DIC, 5 equivalents) in dry dichloromethane (DCM) on ice for 30 min. After evaporation of the DCM and dissolution in DMF, 4-(dimethylamino)-pyridine (0.1 equivalents) was added and the solution was transferred to the Wang resin and stirred for 30 min. Residue couplings for manual SPPS were achieved by stirring the resin at room temperature with the Fmoc protected amino acid (2.5 equivalents), 2-(1H-benzotriazol-1-yl)-1,1,3,3-tetramethyluronium hexafluorophosphate (HBTU, 2.4 equivalents, 0.5 M in DMF) and diisopropylethylamine (DIPEA, 5 equivalents). Deprotection was carried out with piperidine (20% v/v in DMF, 3 × 5 min). Automated syntheses were carried out on a Liberty Blue Microwave Peptide Synthesizer (CEM) using DIC (0.25 M in DMF) and Oxyma (0.5 M in DMF) for coupling of amino acids (5 equivalents) under microwave irradiation for 4 min at 90°C. N-terminal formylation of the effector peptide was achieved with p-nitrophenylformate (3 equivalents) in DMF for 3 h. ISEr effector-PEG_27_ scaffolds were synthesized as described previously (Conibear et al., 2018a) by coupling Fmoc-PEG_27_-OH on the side chains of the two lysine residues: After removal of the 4-methyltrityl (Mtt) side-chain protecting groups with DCM/triisopropyl silane(TIPS)/trifluoroacetic acid(TFA) 98:1:1 (6 × 5 min), Fmoc-PEG_27_-OH (2.75 equivalents) was coupled in DMF/ACN (6:4 v/v) with HATU (2.4 equivalents) and DIPEA (5 equivalents) overnight. Peptides were cleaved from the resin with TFA/TIPS/H_2_O/dimethylsulfide 92.5:2.5:2.5:5 for 3 h and then precipitated with cold diethyl ether, isolated by centrifugation and lyophilised. Effector-PEG_27_ scaffolds bearing Aoa residues were cleaved from the resin in the presence of excess Boc_2_-Aoa as a carbonyl scavenger (Mezö et al., 2011).

#### HPLC Methods

Peptides were purified by RP-HPLC on C4 or C18 preparative or semi-preparative HPLC columns using a linear gradient of (ACN + 0.05% TFA) in (ddH_2_O + 0.05% TFA). Purity was monitored by electrospray ionization mass spectrometry (ESI-MS) in positive ion mode and analytical RP-HPLC using a linear gradient from 5 to 65% (ACN + 0.08% TFA) in (ddH_2_O + 0.1% TFA) over 30 min at 1 mL/min with UV detection at 214 and 280 nm.

### CuAAC Ligations

For CuAAC ligations, all solvents were degassed and reactions were carried out under inert gas. The alkyne-bearing effector-PEG_27_ scaffold (1 equivalent) and the azide-bearing binder peptide (2.5 equivalents) were dissolved in DMF. Fresh stock solutions (CuSO_4_: 200 mM in ddH_2_O, TBTA: 100 mM in DMF, and sodium ascorbate: 500 mM in ddH_2_O) were prepared and the CuSO_4_ (6 equivalents) and TBTA (6.5 equivalents) were mixed before addition of the sodium ascorbate solution (10 equivalents). This solution was then added to the peptide solution, giving a final reaction mixture in 1:4 H_2_O/DMF (v/v). The CuAAC ligation was stirred at room temperature under inert gas and monitored by LC-MS. After completion, the reaction mixture was diluted with water and purified by RP-HPLC. CuAAC ligations between C-terminal azide-bearing ISErs (1 equivalent) and alkyne-functionalised Cy7 (Lumiprobe, 1 equivalent) were carried out as for the CuAAC ligations described above.

### Maleimide Ligations

During removal of the S-tButyl cysteine protecting group and maleimide ligations, the peptides were maintained in degassed solutions and flushed with argon to prevent oxidation of the free thiols. For removal of the S-tButyl group, the purified peptide product after CuAAC ligation was dissolved in a TCEP solution (80 mM in water, pH 6.5), flushed with argon and stirred for 30 min. The TCEP was then removed by purification using RP-HPLC. For the maleimide ligation, the peptide bearing a free cysteine thiol was dissolved in ddH_2_O and the maleimide-functionalised dye (1–2 equivalents) was added in DMF to give a final reaction mixture 1:3 DMF/H_2_O. The mixture was stirred in the dark for 1 h, diluted with water and purified by HPLC.

### Oxime Ligations

The aldehyde functionality on the binder peptides was generated by incubating the N-terminal serine-containing peptide with sodium periodate. The N-terminal serine-containing peptide (final concentration 6 mM) was dissolved in imidazole buffer (50 mM, pH 7) and 4 equivalents of NaIO_4_ in water (stock solution 120 mM) was added. The solution was stirred at room temperature for 1-2 h and the reaction monitored by LC-MS. The aldehyde product was purified by RP-HPLC. For the oxime ligation, the aminooxy-bearing ISEr scaffold (final concentration 2 mM) and the aldehyde-bearing peptide (final concentration 2 mM) were dissolved in 1:1 (v/v) ACN/sodium acetate buffer (0.1 M, pH 4.6) and stirred at room temperature for 24 h. The ligation was monitored by LC-MS and the product purified by HPLC.

### Native Chemical Ligations

NCL reactions with thioester-bearing binder peptides were carried out in 6 M guanidine HCl, 200 mM Na_2_HPO_4_, 40 mM TCEP, and 20 mM MPAA, pH 7.0 at room temperature. NCL reactions with SEA-bearing binder peptides were carried out in 6 M guanidine HCl, 200 mM Na_2_HPO_4_, 200 mM TCEP, and 200 mM MPAA, pH 7.2 at 37°C. The cysteine-bearing effector-PEG_27_ scaffold and the thioester-bearing binder peptide (2.5 equivalents) were dissolved in the ligation buffer and then mixed to give final reaction concentrations of ~ 1 and 2 mM, respectively. The reaction was stirred under inert gas and monitored by LC-MS until it reached completion, or no further change was observed.

### Fluorescence Microscopy

Human colorectal adenocarcinoma HT-29 cells were seeded into 8-well microslides. Cells were incubated with MnCl_2_ (4 mM) in culture medium for 1 h and then fixed with paraformaldehyde (4%). Surface receptors were blocked with bovine serum albumin and then stained in turn with Oregon Green 488 phalloidin (Invitrogen), DAPI and either the fluorescently labeled ISEr (Cy5-labeled integrin α_v_β_6_-targeting ISEr, 374 pM, 647 nm) or an anti-human integrin β_6_-allophycocyanin labeled antibody (~ 267 pM, R&D Systems). After washing with PBS and fixing with paraformaldehyde (4%), cells were imaged using a Zeiss LSM710 confocal laser scanning microscope.

## Data Availability

The datasets generated for this study are available on request to the corresponding author.

## Author Contributions

AC, KT, and CB designed experiments. AC, KT, and NG carried out experiments. AC, KT, and NG analyzed data. AC wrote the paper. AC, KT, NG, and CB edited the paper and approved the submitted manuscript.

### Conflict of Interest Statement

The authors declare that the research was conducted in the absence of any commercial or financial relationships that could be construed as a potential conflict of interest.
