# Supplementary material for: Multifunctional Scaffolds for Assembling Cancer-Targeting Immune Stimulators Using Chemoselective Ligations
Source: Front Chem. 2019 Mar 6;7:113. doi: 10.3389/fchem.2019.00113 (PMC6414710; doi:10.3389/fchem.2019.00113)
Supplement: Supplementary file 1 [file Data_Sheet_1.PDF]

Supporting Information

Multifunctional scaffolds for assembling cancer-targeting immune stimulators using chemoselective ligations

Running Title: chemoselective ligation tools for peptide functionalisation

Frontiers in Chemistry

Special issue on Folded Synthetic Peptides for Biomedical Applications

Anne C. Conibear[1*], Karine Thewes, Nadja Groysbeck and Christian F. W. Becker

[1] Institute of Biological Chemistry, Faculty of Chemistry, University of Vienna, Währinger Straße 38, 1090 Vienna, Austria.

[*]Corresponding author:

Anne C. Conibear

Institute of Biological Chemistry, Faculty of Chemistry, University of Vienna, Währinger Straße 38, 1090 Vienna, Austria

E-mail: anne.conibear@univie.ac.at

Phone: + 43-1-4277-70514

Fax: + 43-1-4277-9705

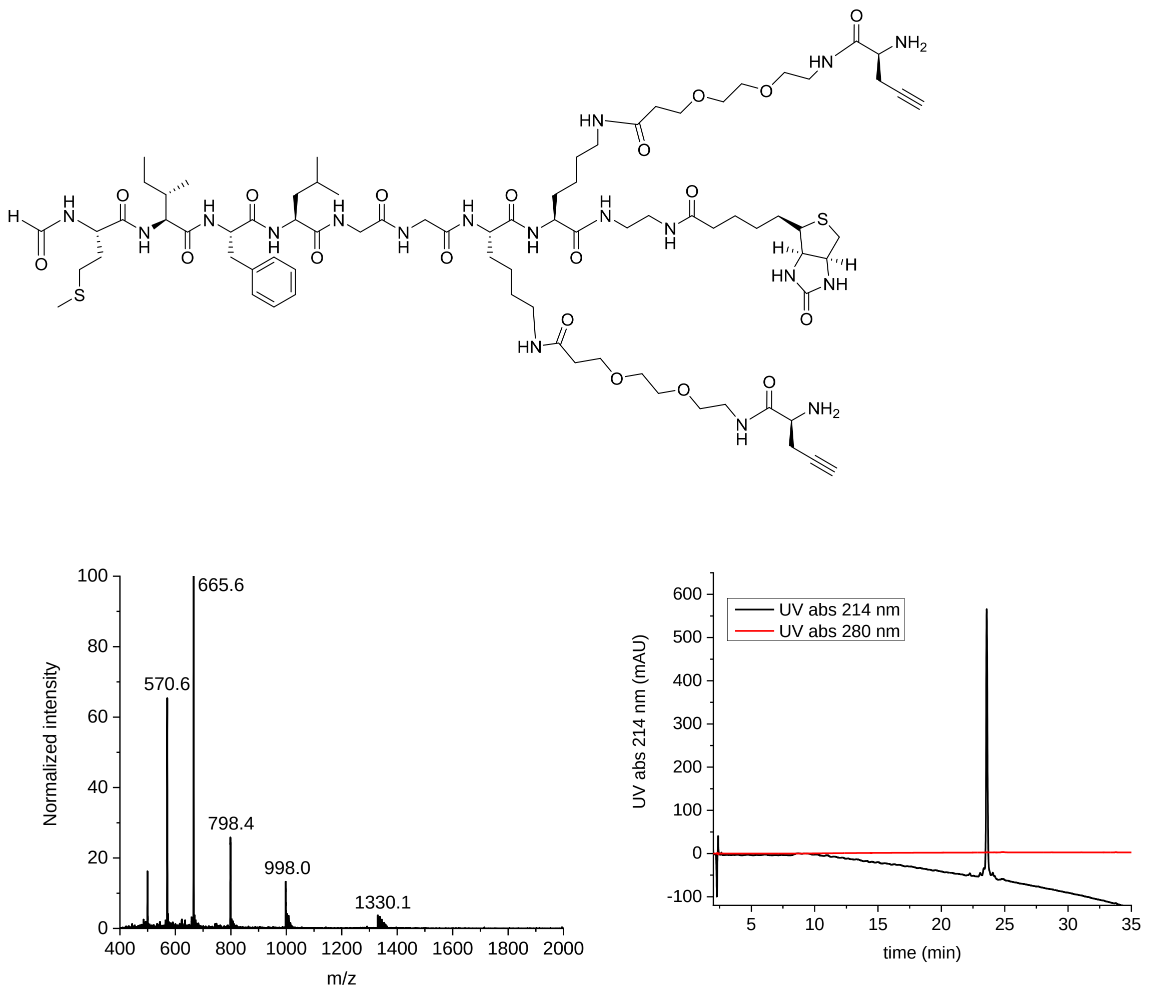

Figure S1. Structure, MS and analytical HPLC of effector-PEG$_{27}$ scaffold for modular synthesis using CuAAC. MW$_{calc}$ 3988.8 Da, MW$_{obs}$ 3987.6 Da. Average yield = 22% based on synthesis scale. Approximate time for synthesis, cleavage, purification and lyophilisation = 5 days.

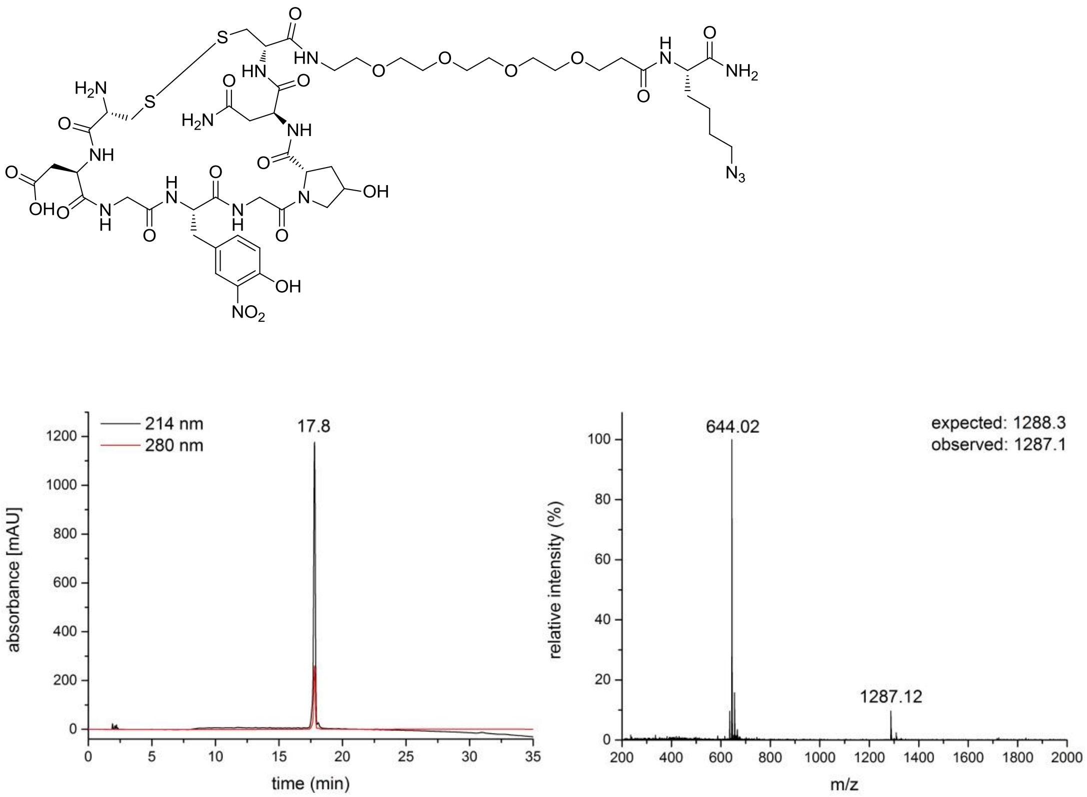

Figure S2. Structure, MS and analytical HPLC of integrin $\alpha_3\beta_1$ binding peptide (B9) for CuAAC. MW_{calc}: 1287.3 Da (ox), MW_{obs}: 1287.1 Da. Yield = 18%, based on synthesis scale. Approximate time for synthesis, cleavage, purification and lyophilisation = 3 days.

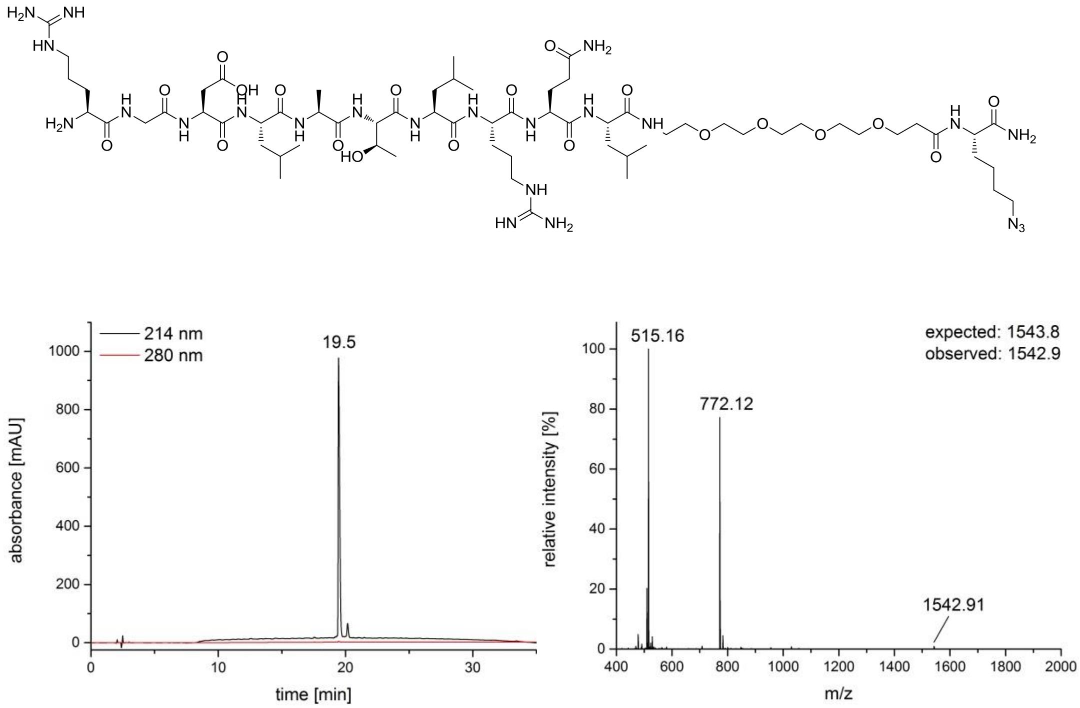

Figure S3. Structure, MS and analytical HPLC of integrin $\alpha_v\beta_6$ binding peptide (B13) for CuAAC. MW_{calc}: 1542.8 Da, MW_{obs}: 1542.9 Da. Yield = 28%, based on synthesis scale. Approximate time for synthesis, cleavage, purification and lyophilisation = 3 days.

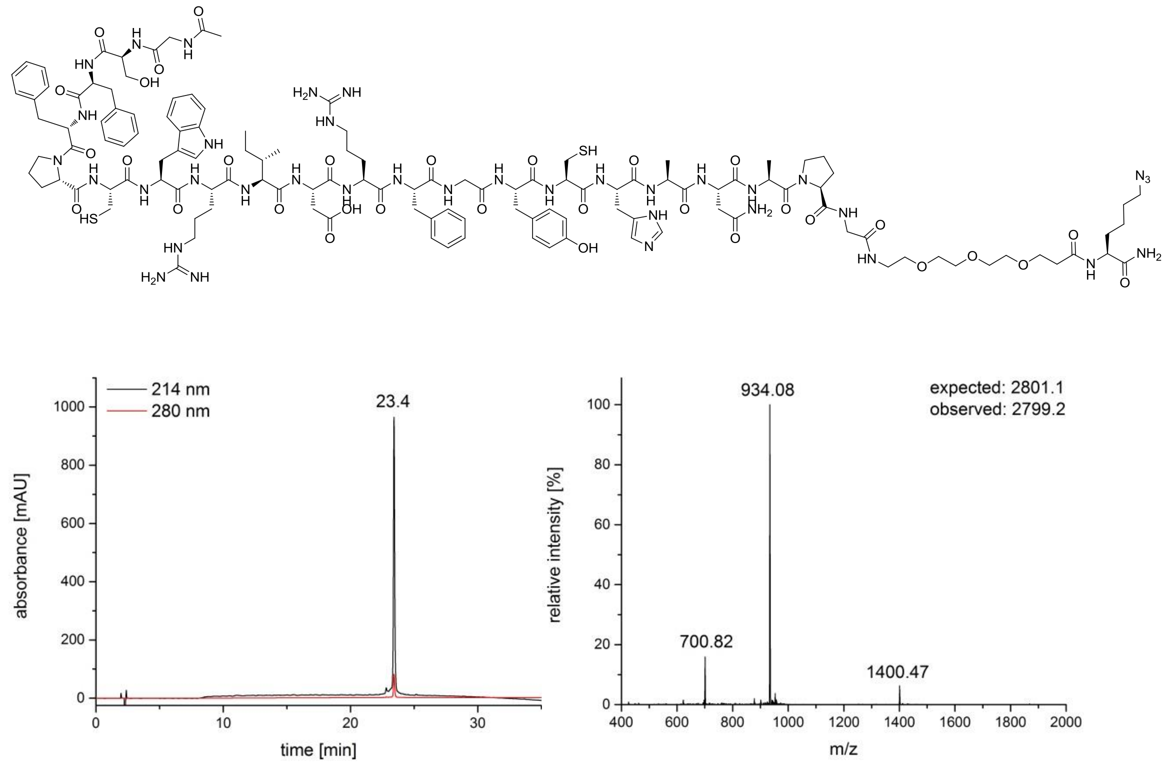

Figure S4. Structure, MS and analytical HPLC of c-Met/HGFR binding peptide (B14) for CuAAC. MW_{calc}: 2800.1 Da, MW_{obs}: 2801.1 Da. Yield = 9%, based on the synthesis scale. Approximate time for synthesis, cleavage, purification and lyophilisation = 4 days.

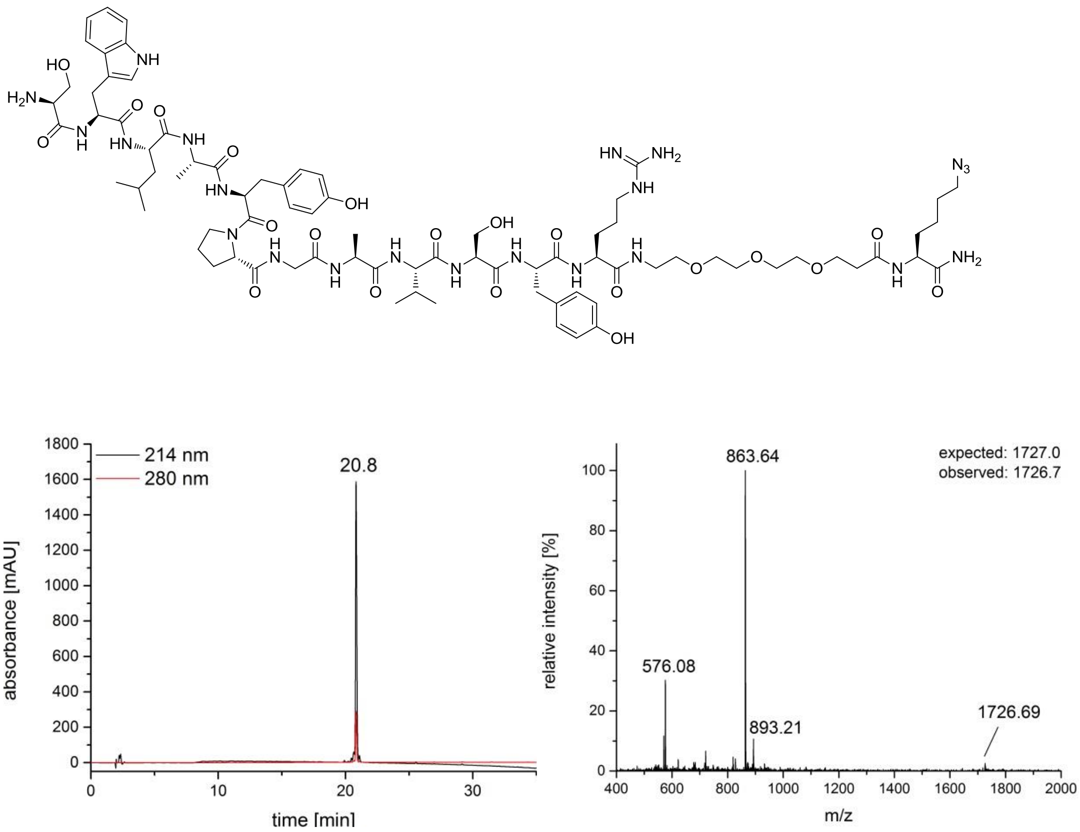

Figure S5. Structure, MS and analytical HPLC of EphA2 binding peptide (B59) for CuAAC. MW_{calc}: 1725.9 Da, MW_{obs}: 1726.7 Da. Yield = 22%, based on the synthesis scale. Approximate time for synthesis, cleavage, purification and lyophilsation = 4 days.

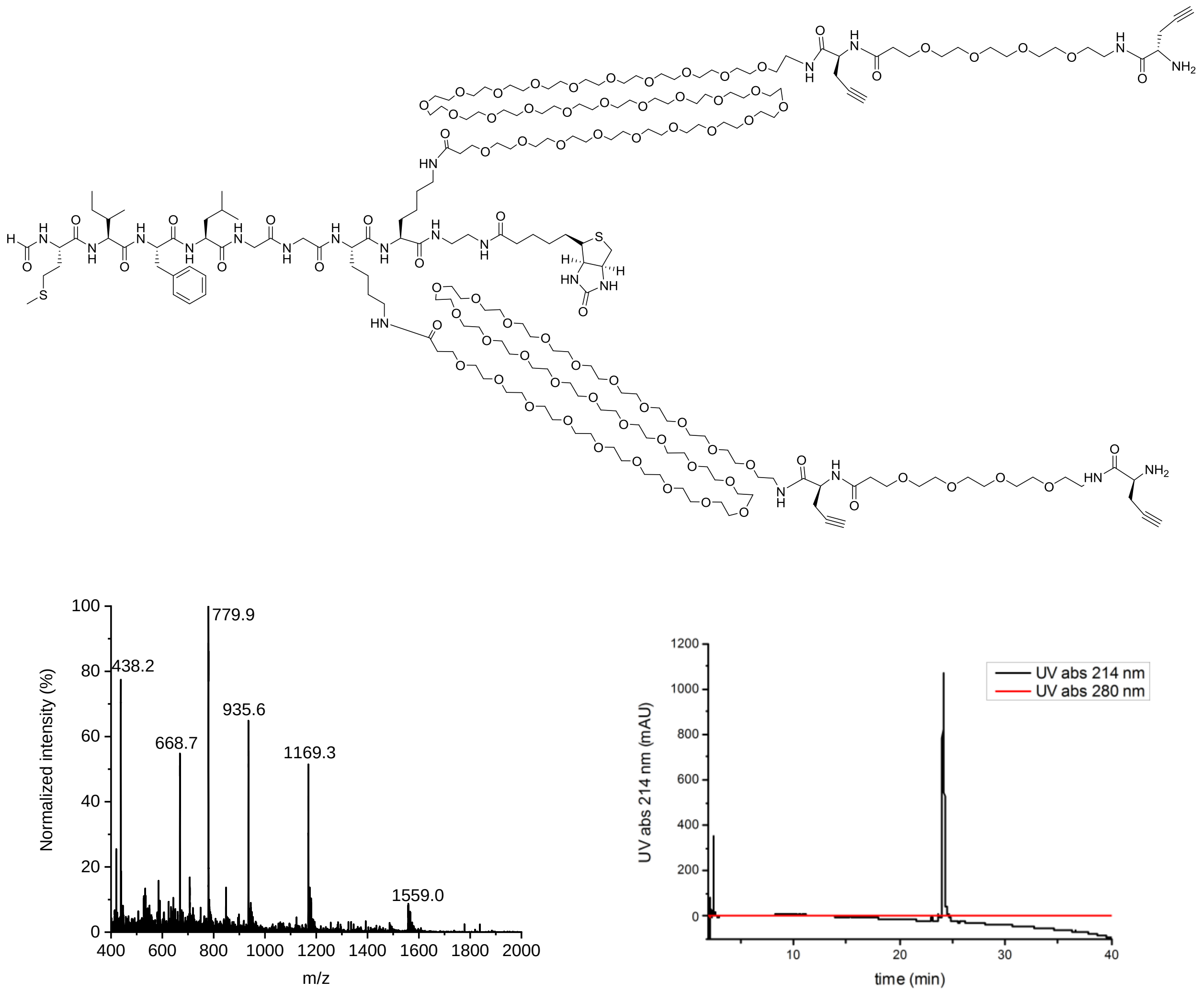

Figure S6. Structure, MS and analytical HPLC of effector-PEG27 scaffold bearing two PEG27 chains and four alkyne moieties. MW_{calc}: 4673.7 Da, MW_{obs}: 4673.0 Da. Yield = 26% based on synthesis scale. Approximate time for synthesis, cleavage, purification and lyophilisation = 5 days.

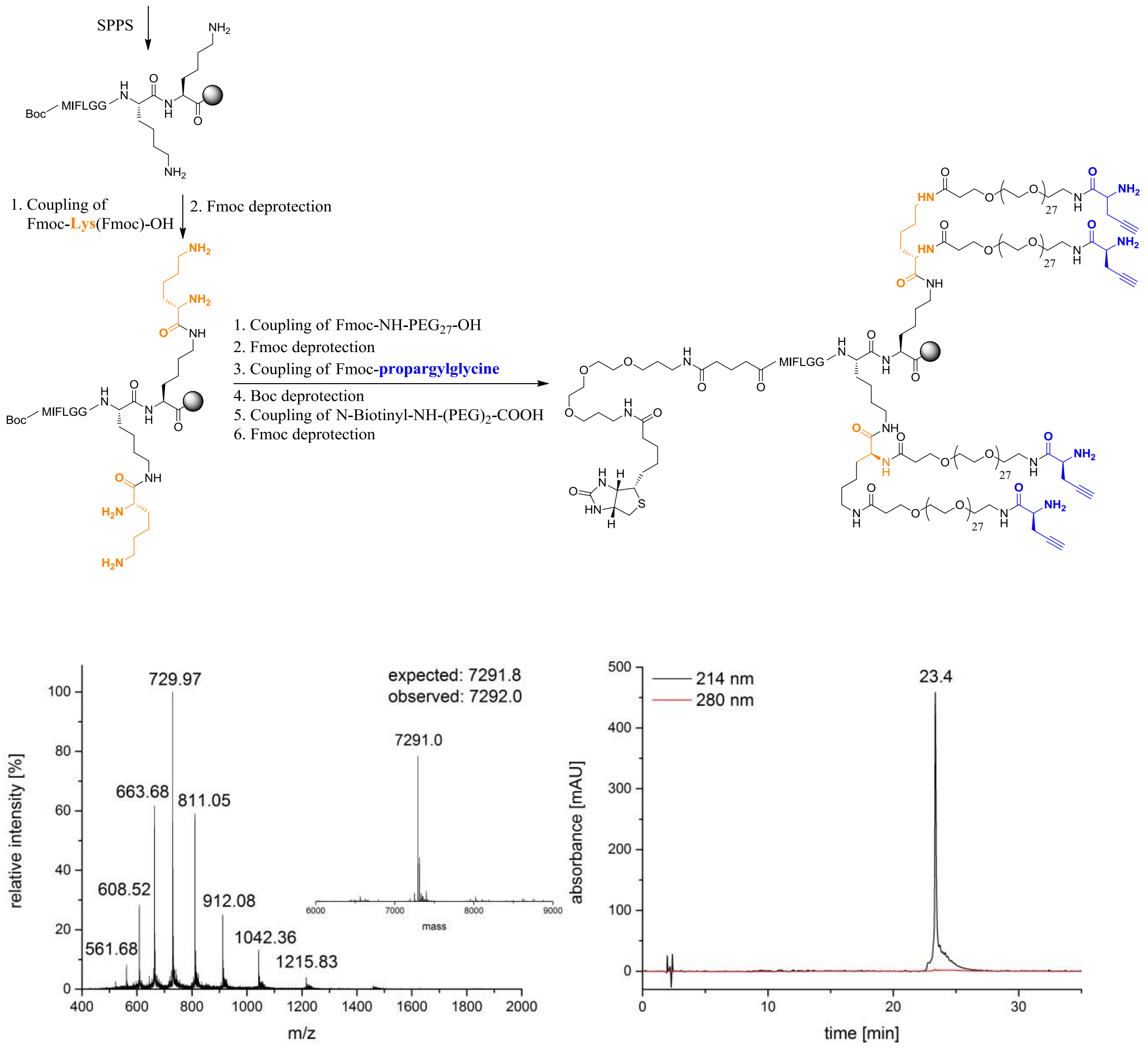

Figure S7. Synthesis, structure, MS and analytical HPLC of effector-PEG27 scaffold bearing four PEG27 chains and four alkyne moieties. MW_{calc}: 7291.8 Da, MW_{obs}: 7292.0 Da. Yield = 0.5% based on the synthesis scale. Approximate time for synthesis, cleavage, purification and lyophilisation = 5 days.

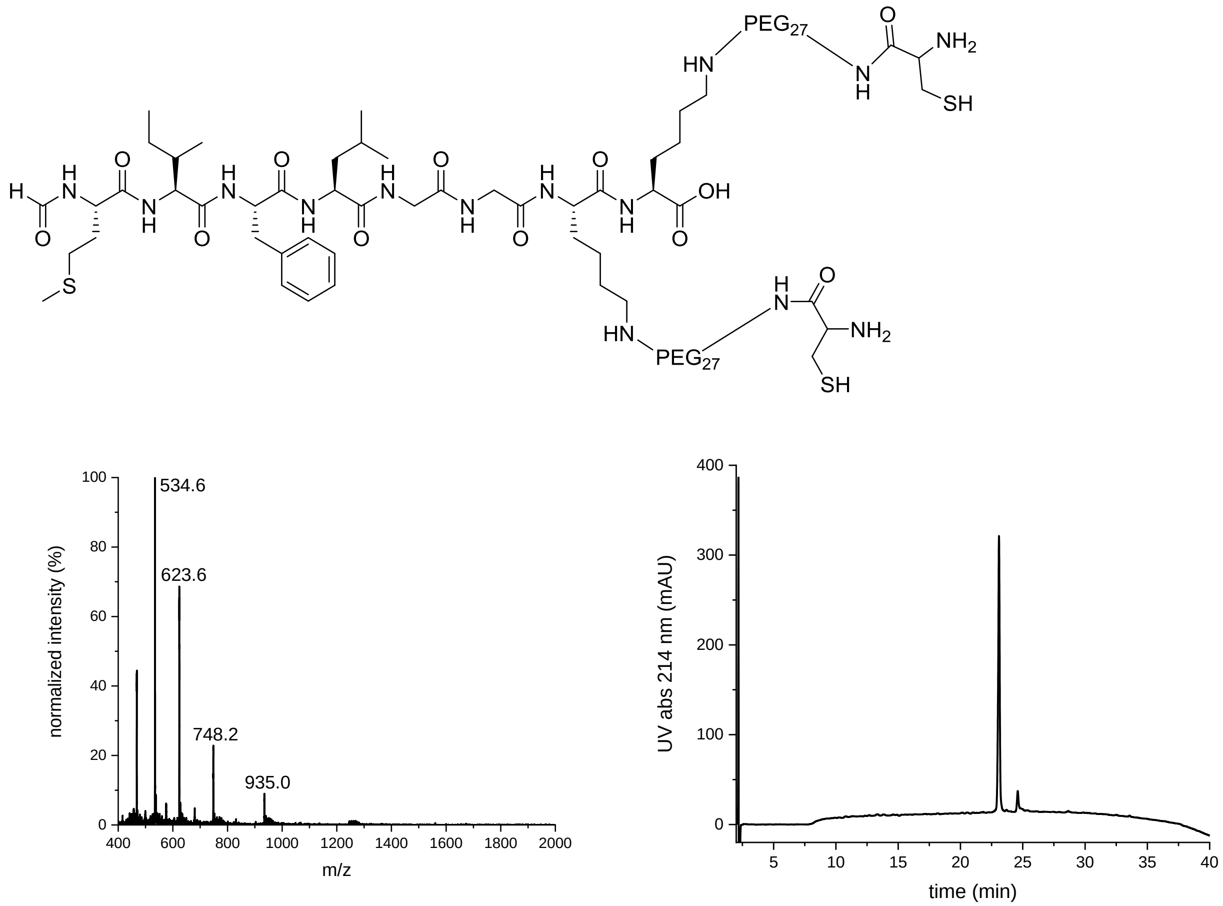

Figure S8. Structure, MS and analytical HPLC of effector-PEG27 scaffold bearing cysteine residues for NCL. MW_{calc}: 3736.5 Da, MW_{obs}: 3737.5 Da. Yield = 14% based on synthesis scale. Approximate time for synthesis, cleavage, purification and lyophilisation = 4 days.

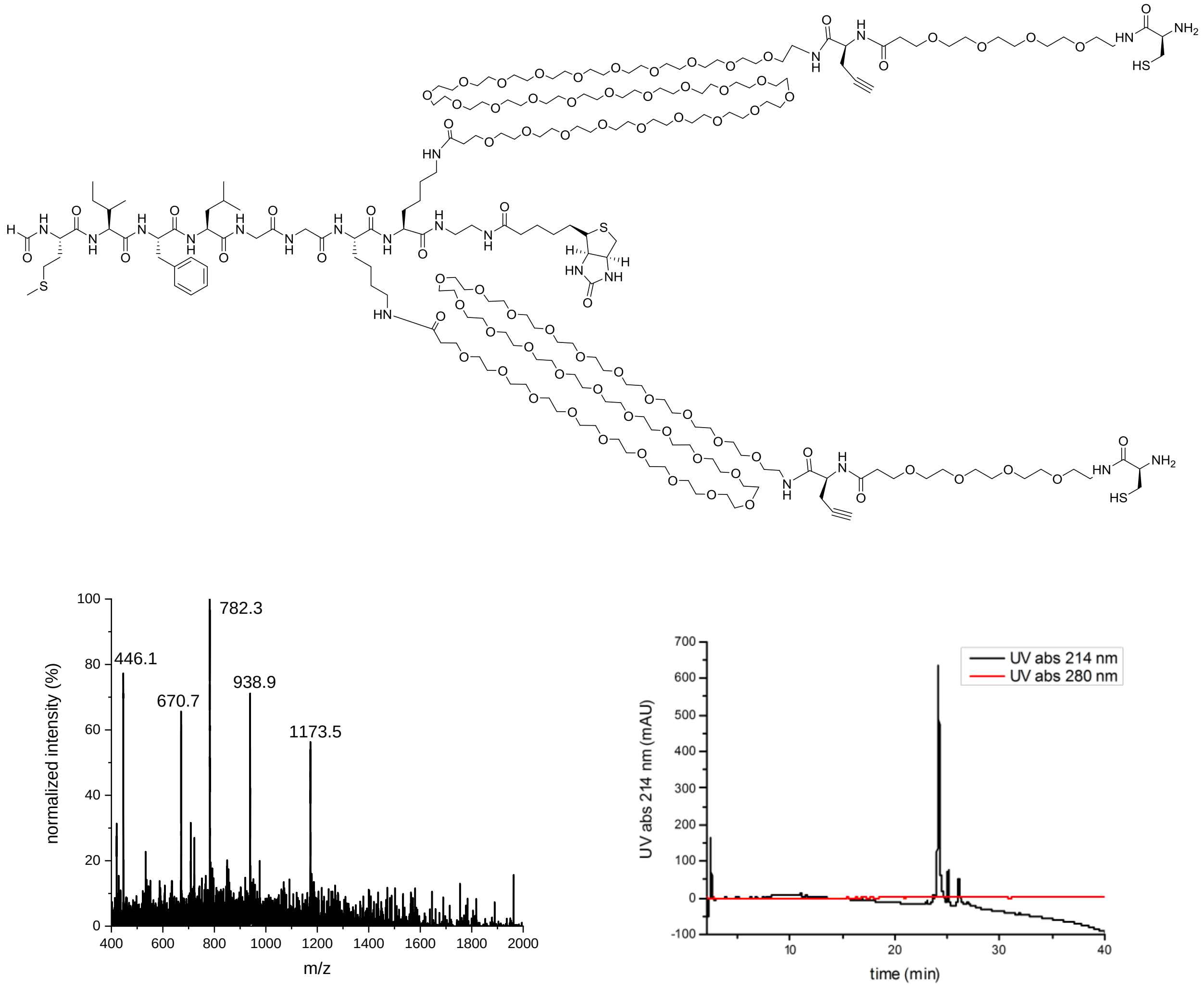

Figure S9. Structure, MS and analytical HPLC of effector-PEG$_{27}$ scaffold bearing handles for both CuAAC and NCL. MW_{calc}: 4689.7 Da, MW_{obs}: 4689.0 Da. Yield = 20% based on synthesis scale. Approximate time for synthesis, cleavage, purification and lyophilisation = 5 days.

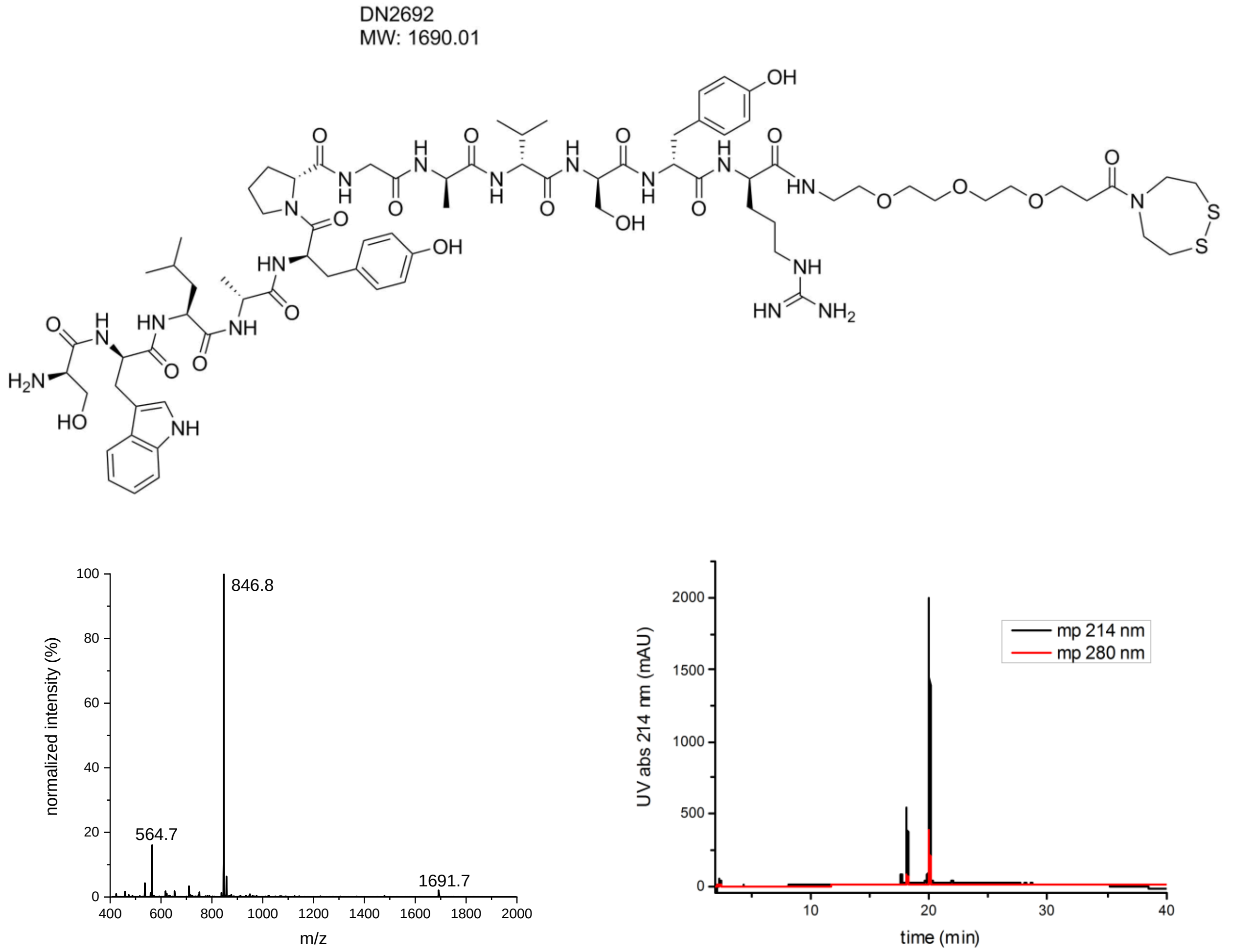

Figure S10. Structure, MS and analytical HPLC of EphA2 binder peptide with SEA thioester precursor for NCL. MW_{calc}: 1690.0 Da, MW_{obs}: 4689.0 Da. The two peaks in the analytical HPLC spectrum are caused by the two isomers of the SEA moiety formed by N-S acyl transfer. Yield = 24% based on synthesis scale. Approximate time for resin preparation, synthesis, cleavage, purification and lyophilisation = 5 days.

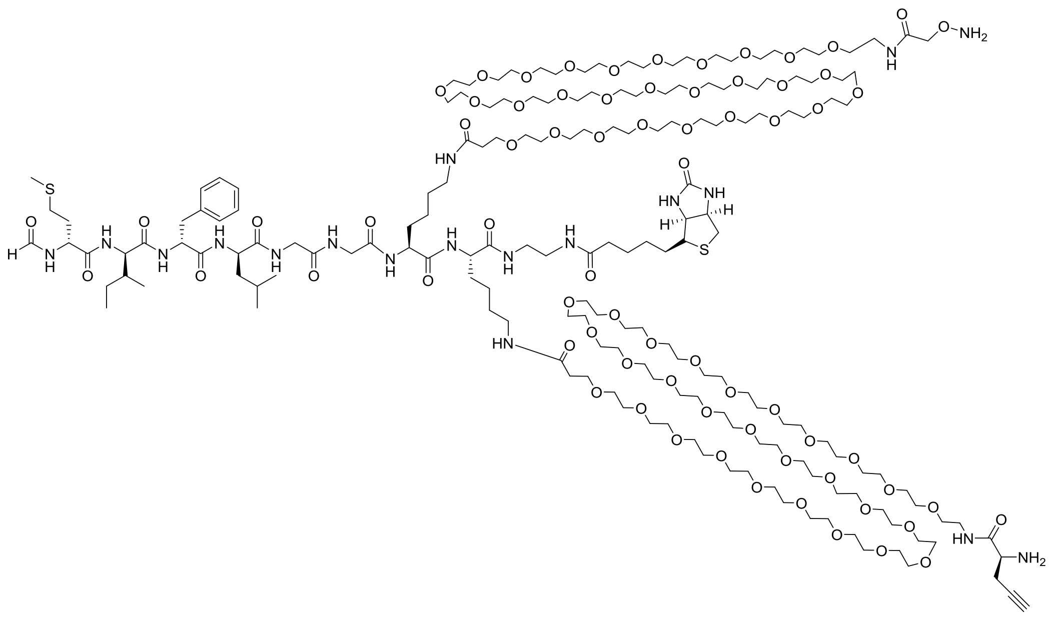

Figure S11. Structure of effector-PEG27 scaffold bearing handles for both CuAAC and oxime ligations to give bivalent bispecific ISErs. MW_{calc}: 3966.8 Da, MW_{obs}: 3967.5 Da.

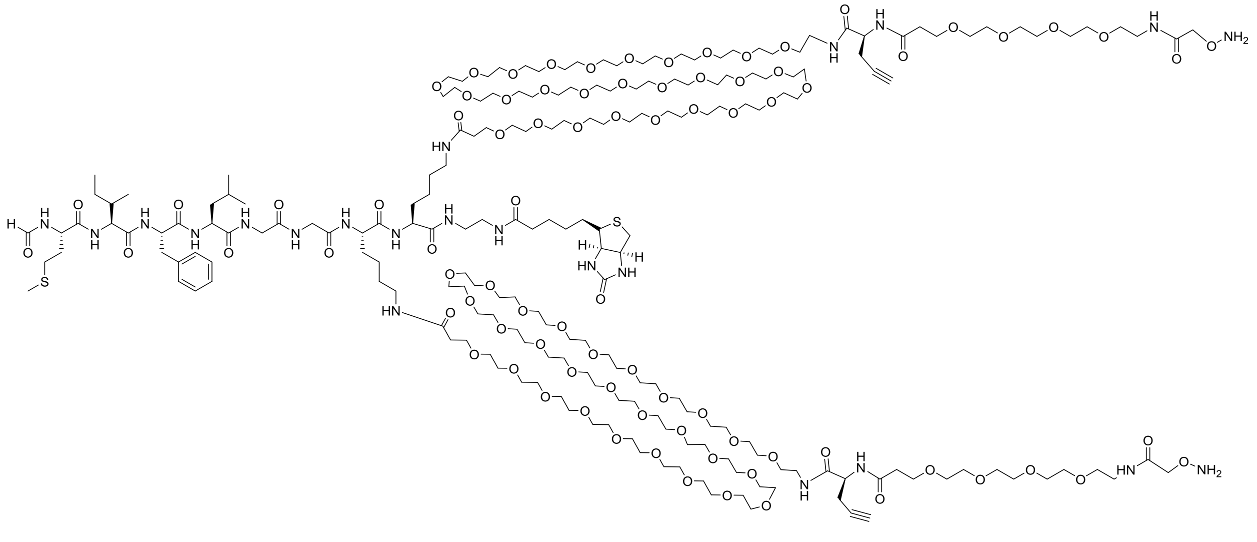

Figure S12. Structure of effector-PEG27 scaffold bearing handles for both CuAAC and oxime ligations to give tetravalent bispecific ISErs. MW_{calc}: 4629.6 Da, MW_{obs}: 4628.0 Da.

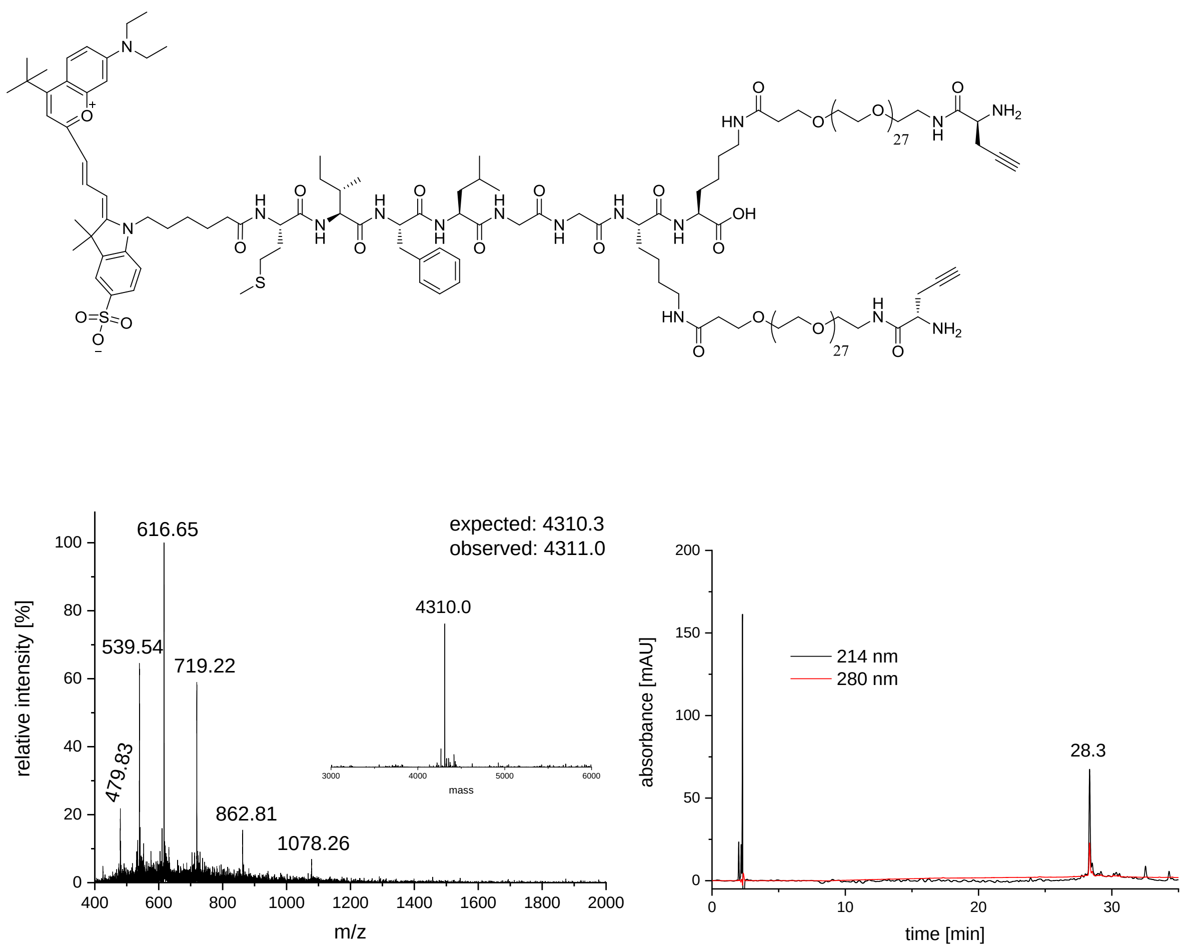

Figure S13. Structure, MS and analytical HPLC of DY680-labelled effector-PEG27 scaffold for CuAAC ligation. MW_{calc}: 4309.3 Da, MW_{obs}: 4310.0 Da. Yield = 12% based on the synthesis scale. Approximate time for synthesis, dye coupling, cleavage, purification and lyophilisation = 5 days.

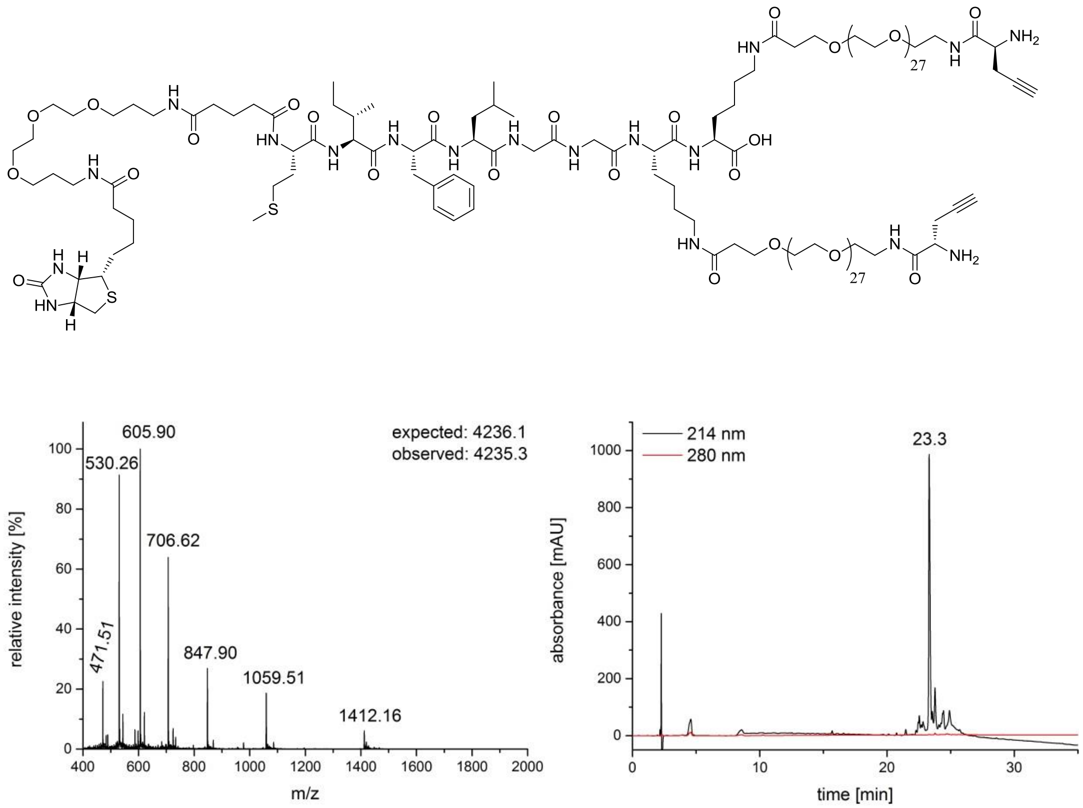

Figure S14. Structure, MS and analytical HPLC of N-terminal biotinylated effector-PEG27 scaffold for CuAAC ligation. MW_{calc}: 4235.1 Da, MW_{obs}: 4235.3 Da. Yield = 11% based on the synthesis scale. Approximate time for synthesis, cleavage, purification and lyophilisation = 5 days.

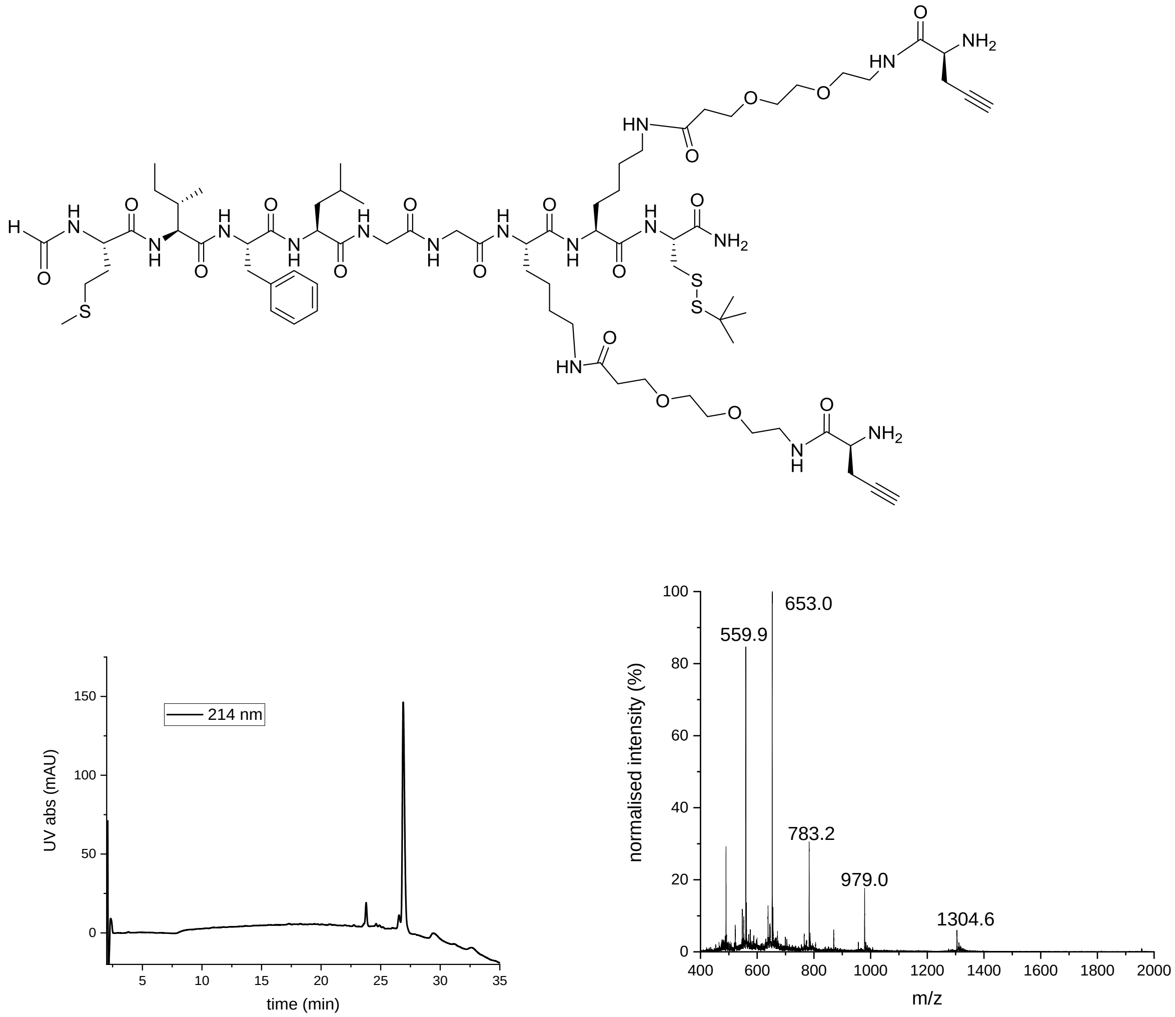

Figure S15. Structure, MS and Analytical HPLC of effector-PEG$_{27}$ scaffold with C-terminal cysteine (S-tBu-protected) for maleimide ligation. MW_{calc}: 3910.8 Da, MW_{obs}: 3912.0 Da. Yield = 10% based on synthesis scale. Approximate time for synthesis, cleavage, purification and lyophilisation = 5 days.

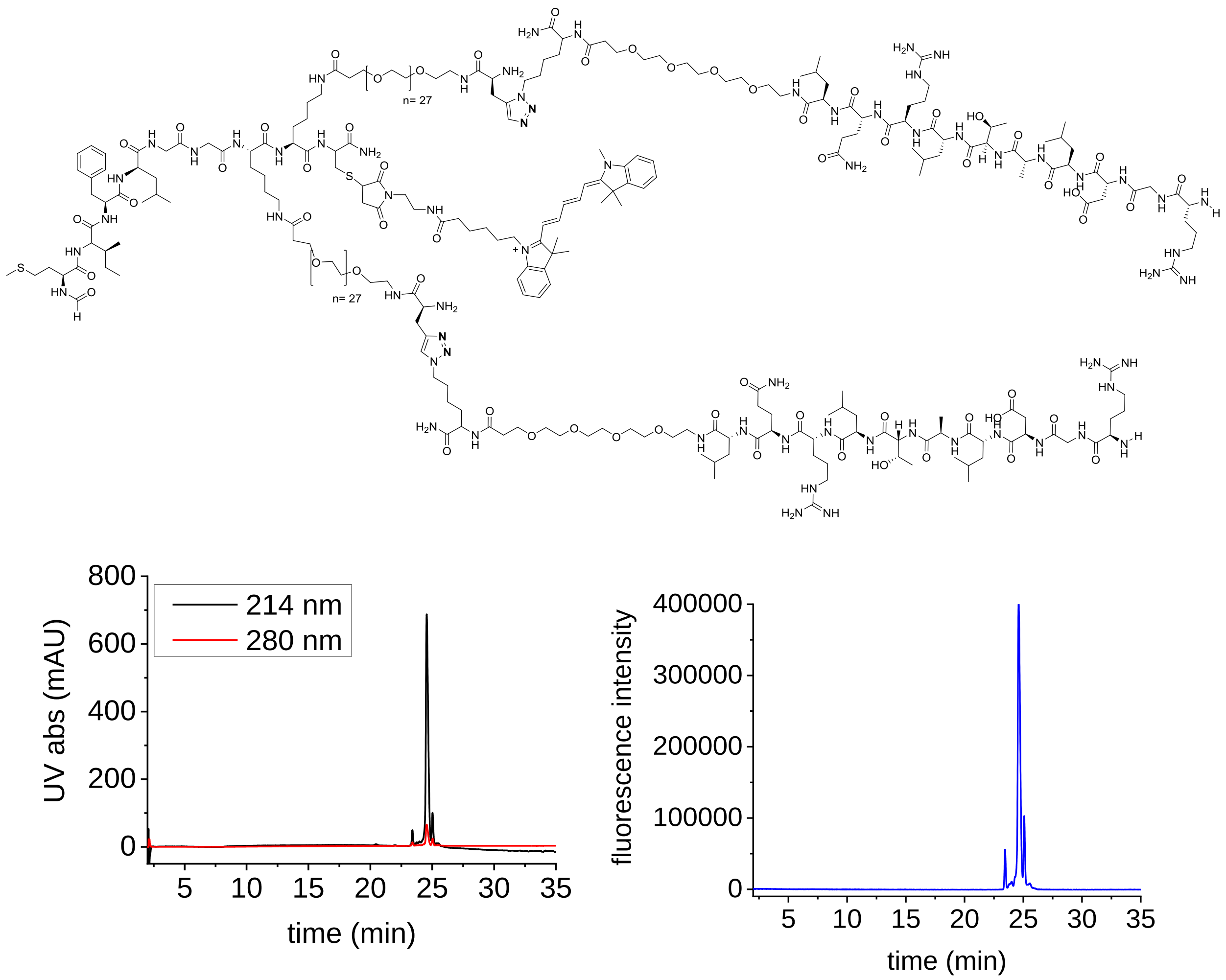

Figure S16. Structure of integrin $\alpha_v\beta_6$ – targeting ISEr labelled with Cy5 maleimide and analytical HPLC traces with detection at 214 and 280 nm (left) and 650 nm (right). Isolated yields after click reaction = 41%, after StBu removal = 88%, and after Cy5 maleimide coupling = 48%. Overall yield of final product = 2%, based on synthesis scale of the scaffold. Approximate time for synthesis of both components, ligation reactions and all purification and lyophilisation steps = 10 days.

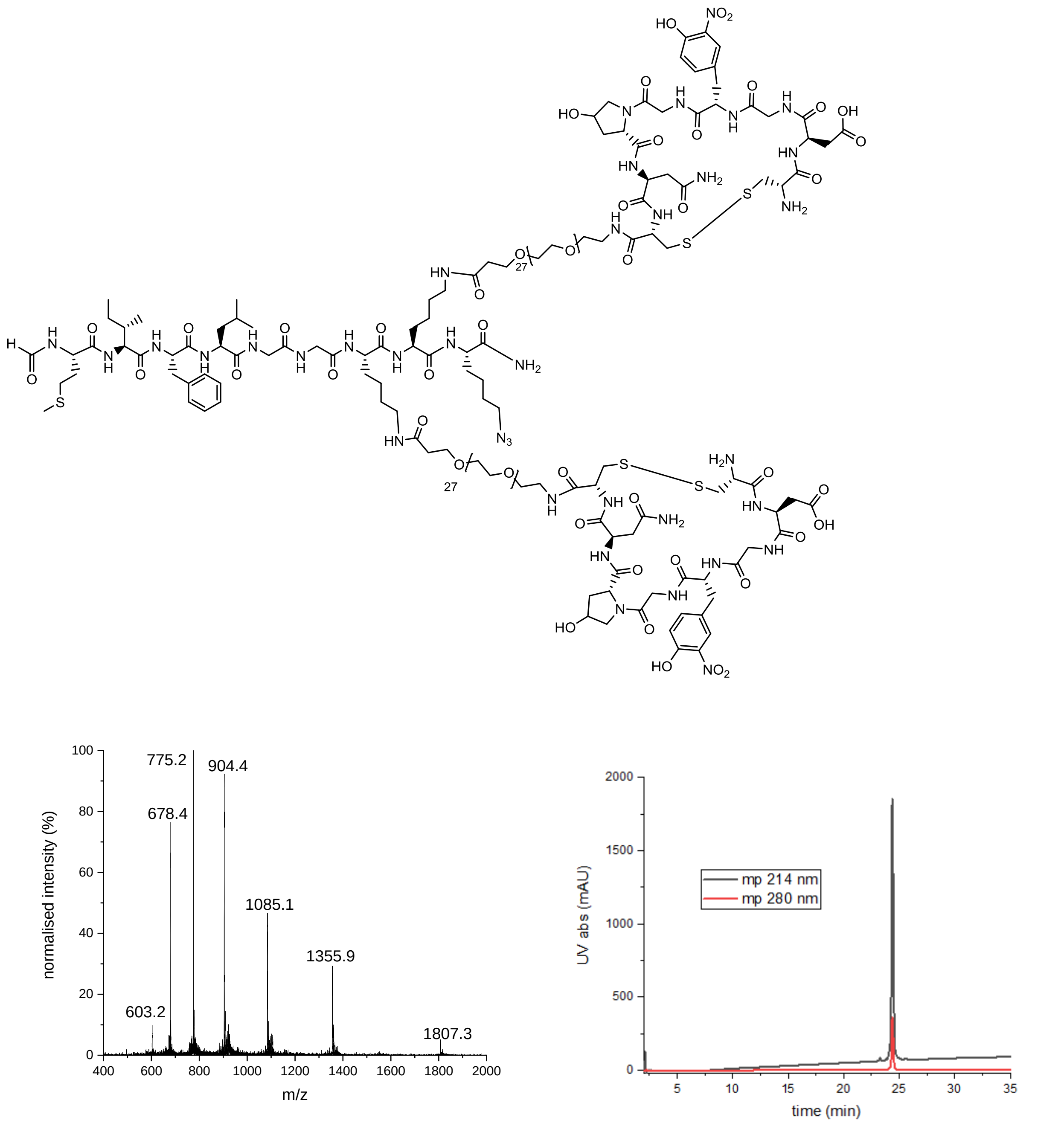

Figure S17. Structure, MS and analytical HPLC of integrin α3β1 binding ISEr with C-terminal azidolysine for CuAAC ligation. MW_{calc}: 5421.2 Da, MW_{obs}: 5419.5 Da. Yield = 9%, based on synthesis scale. Approximate time for synthesis, cleavage, purification and lyophilisation = 6 days.

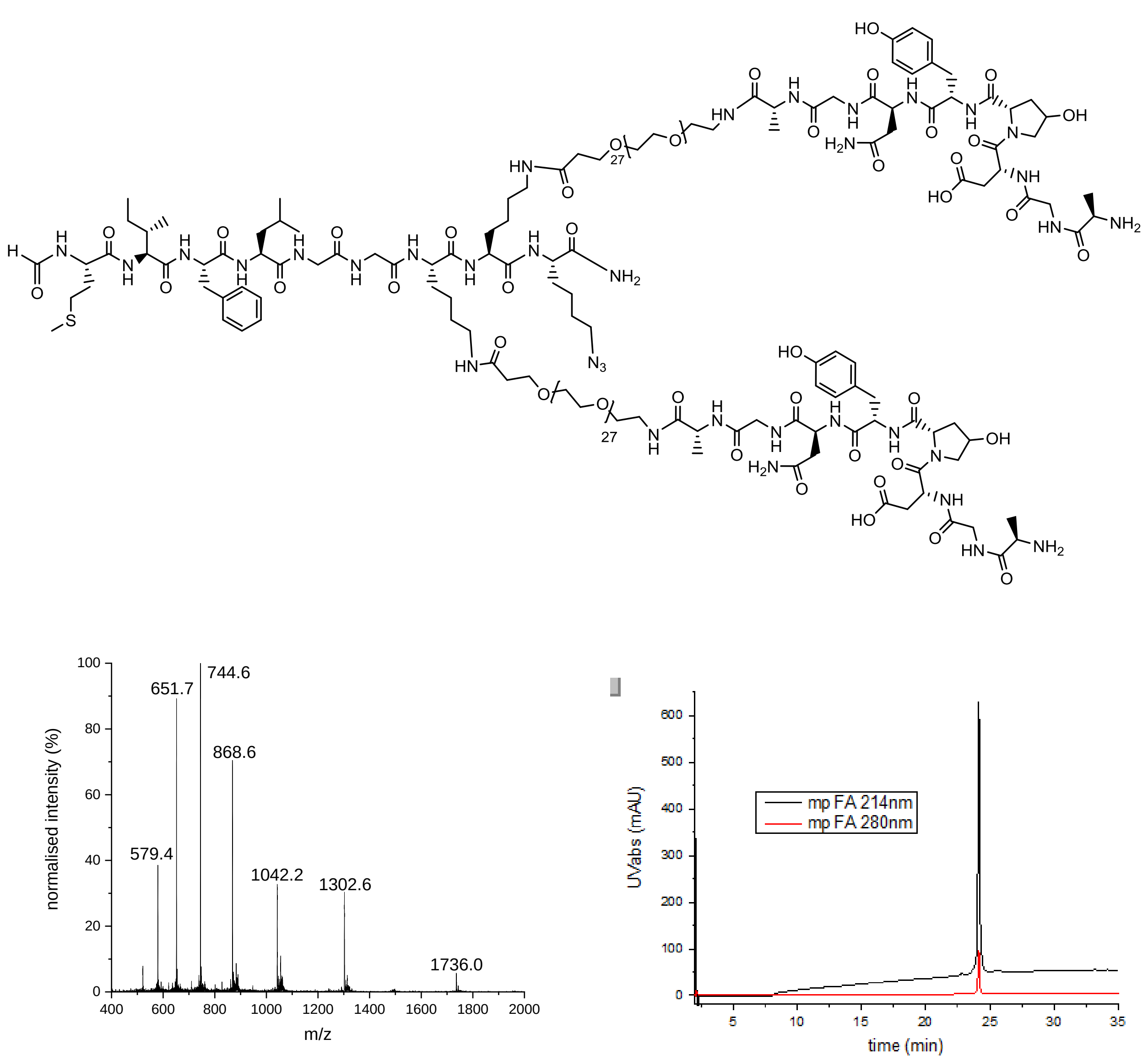

Figure S18. Structure, MS and analytical HPLC of scrambled integrin α3β1 binding ISEr with C-terminal azidolysine for CuAAC ligation. MW_{calc}: 5207.0 Da, MW_{obs}: 5205.3 Da. Yield = 23% based on synthesis scale. Approximate time for synthesis, cleavage, purification and lyophilisation = 6 days.

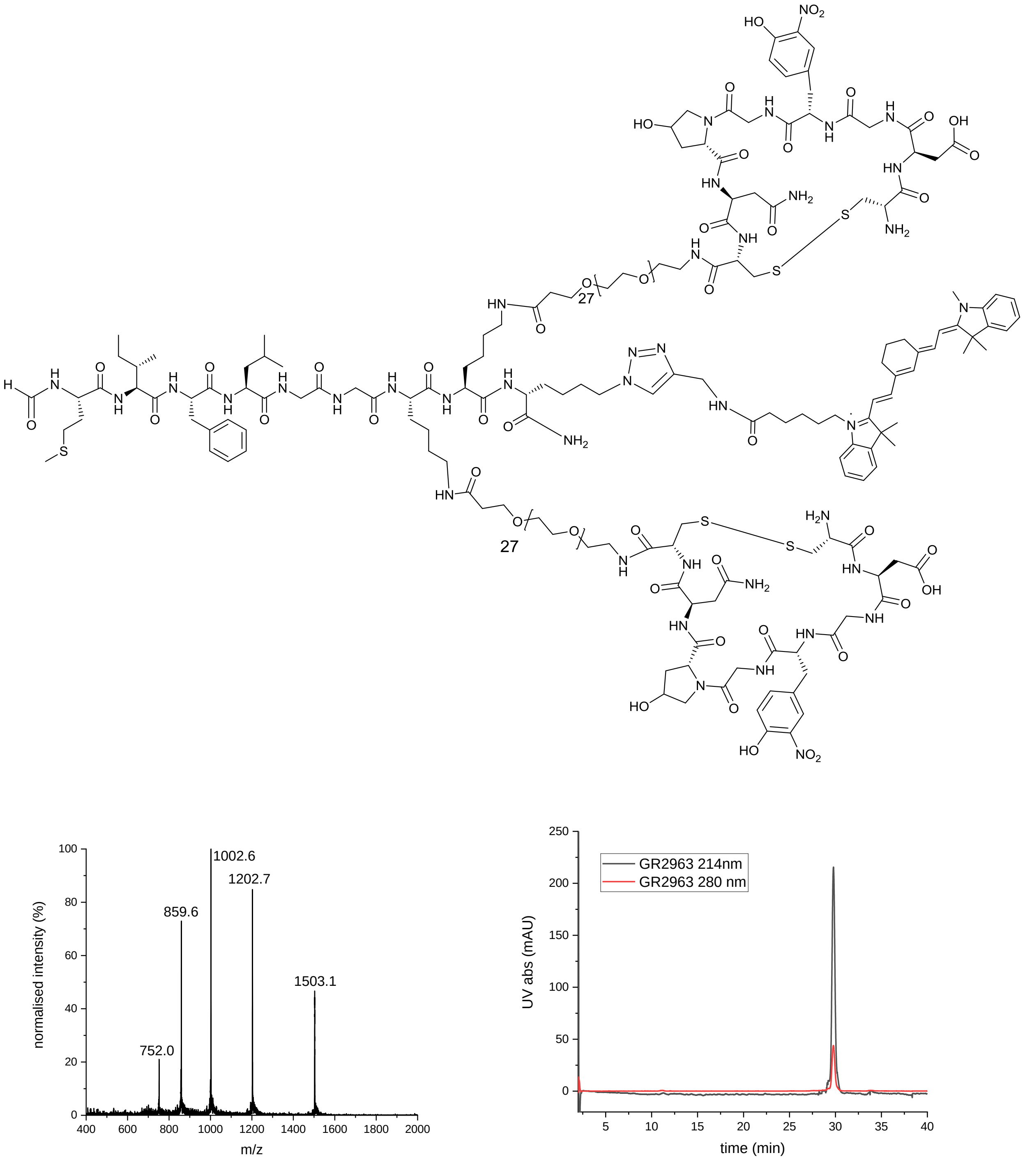

Figure S19. Structure, MS and analytical HPLC of integrin $\alpha_3\beta_1$ binding ISEr ligated to Cy7. MW_{calc}: 6008.0 Da, MW_{obs}: 6009.6 Da. Yield = 27% from click reaction, isolated yield. Approximate time for synthesis, click reaction and purification steps = 9 days.

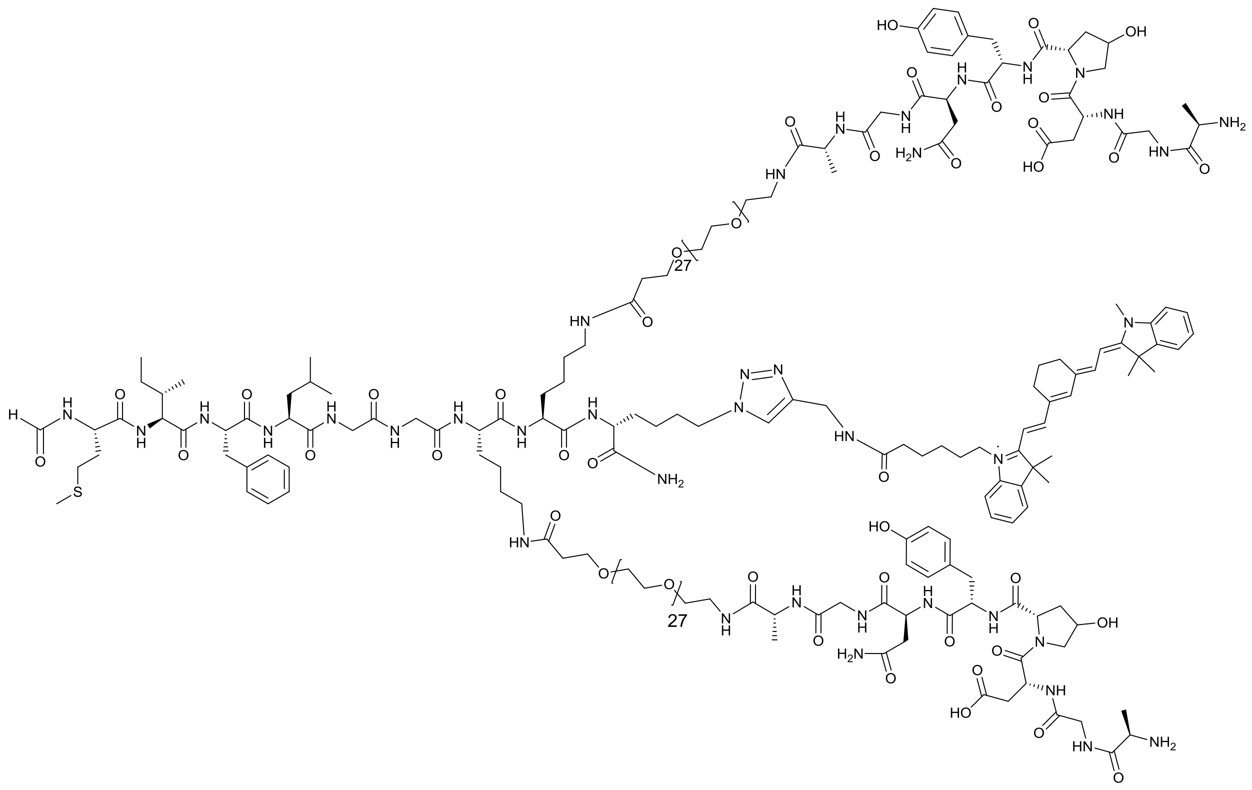

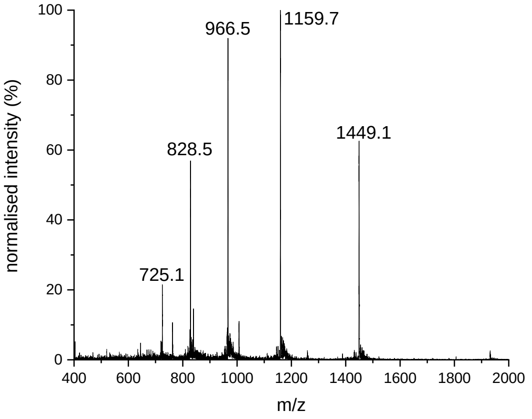

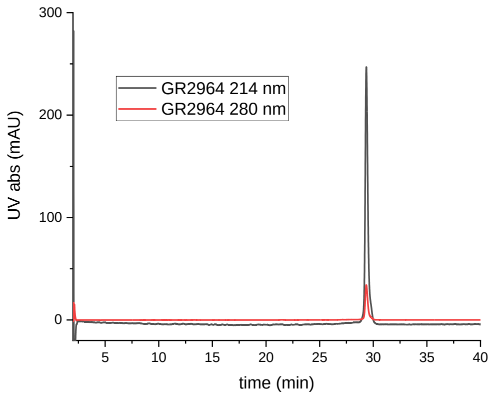

Figure S20. Structure, MS and analytical HPLC of scrambled integrin $\alpha_3\beta_1$ binding ISEr ligated to Cy7. MWcalc: 5793.8 Da, MWobs: 5793.2 Da. Yield = 27% from click reaction, isolated yield. Approximate time for synthesis, click reaction and purification steps = 9 days.
